# Photosynthesis-driven interactions in the phycosphere enhance bacterial extracellular superoxide production

**DOI:** 10.1093/ismejo/wrag175

**Published:** 2026-07-05

**Authors:** Xi Wang, Baiwen Ma, Hongwei Yu, Lin Su, Jing Qi, Zhugen Yang, Chengzhi Hu, Jiuhui Qu

**Affiliations:** Key Laboratory of Environmental Aquatic Chemistry, State Key Laboratory of Regional Environment and Sustainability, Research Center for Eco-Environmental Sciences, Chinese Academy of Sciences, Beijing 100085, China; College of Resources and Environment, University of Chinese Academy of Sciences, Beijing 100049, China; Key Laboratory of Environmental Aquatic Chemistry, State Key Laboratory of Regional Environment and Sustainability, Research Center for Eco-Environmental Sciences, Chinese Academy of Sciences, Beijing 100085, China; College of Resources and Environment, University of Chinese Academy of Sciences, Beijing 100049, China; Key Laboratory of Environmental Aquatic Chemistry, State Key Laboratory of Regional Environment and Sustainability, Research Center for Eco-Environmental Sciences, Chinese Academy of Sciences, Beijing 100085, China; College of Resources and Environment, University of Chinese Academy of Sciences, Beijing 100049, China; Department of Biochemistry, School of Biological and Behavioural Sciences, Queen Mary University of London, Mile End Road, London E1 4NS, United Kingdom; Key Laboratory of Environmental Aquatic Chemistry, State Key Laboratory of Regional Environment and Sustainability, Research Center for Eco-Environmental Sciences, Chinese Academy of Sciences, Beijing 100085, China; College of Resources and Environment, University of Chinese Academy of Sciences, Beijing 100049, China; Faculty of Engineering and Applied Sciences, Cranfield University, Cranfield MK43 0AL, United Kingdom; Key Laboratory of Environmental Aquatic Chemistry, State Key Laboratory of Regional Environment and Sustainability, Research Center for Eco-Environmental Sciences, Chinese Academy of Sciences, Beijing 100085, China; College of Resources and Environment, University of Chinese Academy of Sciences, Beijing 100049, China; Key Laboratory of Environmental Aquatic Chemistry, State Key Laboratory of Regional Environment and Sustainability, Research Center for Eco-Environmental Sciences, Chinese Academy of Sciences, Beijing 100085, China; College of Resources and Environment, University of Chinese Academy of Sciences, Beijing 100049, China

**Keywords:** algal-bacterial interactions, extracellular superoxide, phycosphere, biogenic reactive oxygen species, siderophore-associated Fe competition, contaminant transformation

## Abstract

Extracellular superoxide generated by heterotrophic bacteria influences aquatic redox transformations, yet its regulation within the phycosphere remains poorly understood. Here, we show that the cyanobacterium *Microcystis aeruginosa* enhances extracellular superoxide production by *Pseudomonas* sp. QJX-1 under illumination. Cocultivation increased extracellular superoxide production approximately three-fold over bacterial monoculture, with maximum enhancement during carbon starvation. Membrane separation localized detectable superoxide production to the bacterial chamber, indicating that diffusible algal products stimulated bacterial superoxide production. Mechanistically, illuminated cocultures showed 20.4% higher photocurrent than algal monocultures and accumulated the highest extracellular NADP(H) levels, indicating intensified photosynthesis-associated extracellular redox activity. Reduced nicotinamide cofactors stimulated diphenyleneiodonium-sensitive QJX-1-associated superoxide production, consistent with an oxidoreductase-associated route for one-electron reduction of molecular oxygen. In parallel, coculture accumulated catecholate siderophores, consistent with intensified microscale Fe competition; this siderophore-enhanced response required metabolically active bacterial cells and was attenuated by respiratory-chain inhibition. Representative extracellular redox-active and algal photoactive components further enhanced the bacterial response, with cytochrome *c* and chlorophyll *a* stimulating QJX-1-associated superoxide production under defined conditions. Functionally, during the carbon-starvation interval coinciding with peak superoxide production, the apparent sulfamethoxazole removal rate constant in coculture was 2.6 times that observed for QJX-1 alone. Moreover, quenching extracellular reactive oxygen species (ROS) impaired metabolic activity under diverse chemical stressors and reduced carbon, nitrogen, and phosphorus substrate utilization. Collectively, these findings identify photosynthesis-dependent phycosphere interactions as regulators of bacterial superoxide production, linking biogenic ROS to contaminant transformation, chemical-stress resilience, and elemental nutrient turnover.

## Introduction

Reactive oxygen species (ROS) are important redox-active intermediates in aquatic environments, where they influence elemental cycling, nutrient bioavailability, organic matter transformation, and pollutant fate [1–3]. Among them, extracellular superoxide (O_2_^•−^) is one of the most widespread and environmentally consequential ROS in natural waters [4, 5]. Heterotrophic bacteria are now recognized as important biological sources of extracellular O_2_^•−^, and its production is increasingly understood not simply as a byproduct of oxidative stress, but as a physiologically regulated extracellular process associated with membrane-linked oxidoreductase activity [6–8]. This pattern suggests that extracellular O_2_^•−^ production is not merely an uncontrolled metabolic byproduct, but may be maintained because it serves physiologically relevant functions. Indeed, extracellular O_2_^•−^ has been linked to growth-related physiology in heterotrophic marine bacteria and to photophysiological function in phototrophic microorganisms [6, 7, 9, 10]. Consistent with this view, bacterially produced extracellular O_2_^•−^ also contributes to the redox transformation of elements such as Fe, Mn, Sb, and Hg, regulates trace-nutrient bioavailability, and influences organic matter transformation and oxygen cycling [11–15]. For example, extracellular O_2_^•−^ produced by *Roseobacter* sp. AzwK-3b can drive Mn(II) oxidation and promote the formation of highly reactive biogenic Mn oxides, thereby affecting the environmental fate of trace metals such as Cu, Co, and Ni [14]. Extracellular O_2_^•−^ produced by marine bacteria can also participate in the pre-oxidative transformation of pollutants such as ibuprofen [16]. In addition, sustained dark extracellular O_2_^•−^ production by marine microorganisms may represent a previously underappreciated sink in the marine dissolved oxygen budget [4, 17]. These observations identify extracellular O_2_^•−^ as an important interfacial reactive species linking microbial physiology to redox transformation in natural waters.

Despite growing recognition of its ecological significance, how extracellular O_2_^•−^ production is regulated remains incompletely understood [1]. Current understanding derives largely from isolate-based studies that define its physiological basis and from environmental measurements that resolve its distribution, fluxes, and biogeochemical relevance [4–6, 18]. However, these approaches provide limited insight into how extracellular O_2_^•−^ production is regulated in the microscale habitats where aquatic bacteria actually live and interact, particularly under close metabolic association with phototrophs.

One such habitat is the phycosphere, the microscale zone surrounding algal cells, where heterotrophic bacteria are often highly enriched [19]. Formed by the continuous release of photosynthates, low-molecular-weight metabolites, and other extracellular compounds, the phycosphere is characterized by steep gradients in organic substrates, dissolved oxygen, pH, and trace elements [20–22]. Through the combined effects of algal photosynthesis, nutrient uptake, and sustained metabolism by both algae and bacteria, the phycosphere functions not only as a hotspot of metabolite exchange and bacterial colonization, but also as a dynamic microscale redox interface [23–25]. Extracellular O_2_^•−^ production by heterotrophic bacteria in this environment is therefore unlikely to be determined by bacterial metabolism alone and may instead be systematically reshaped by algal activity and by local chemical heterogeneity [26].

Several lines of evidence suggest that algae can restructure interfacial redox processes in ways relevant to extracellular O_2_^•−^ production. In particular, previous studies have shown that algal–bacterial associations in the phycosphere involve extensive exchange of metabolites and other redox-relevant compounds, with consequences for bacterial gene expression, nutrient acquisition, and interfacial redox chemistry [20, 24]. Photosynthetic microorganisms release low-molecular-weight metabolites, reduced nicotinamide cofactors, and other redox-active compounds into the extracellular milieu [27–30]. In cyanobacterial and related photobioelectrochemical systems, illumination has been associated with extracellular NADPH accumulation and enhanced interfacial redox activity [31, 32]. Reduced nicotinamide cofactors are also closely linked to extracellular O_2_^•−^ generation. NADH stimulates extracellular O_2_^•−^ production by heterotrophic bacteria, whereas NADPH has also been implicated as an electron donor supporting extracellular O_2_^•−^ production in some phototrophic microorganisms [6, 10]. In parallel, metabolite-rich conditions in the phycosphere can alter substrate use and metabolic pathway expression in adjacent bacteria [33, 34]. Algal-derived redox-active mediators and photosensitizing compounds may further enhance or modulate interfacial redox activity and ROS production. For example, photosynthetic pigments released from dead microalgal cells can act as extracellular photoactive components that enhance light-driven redox transformations mediated by bacteria [35]. Together, these observations suggest that algae may influence neighboring bacteria not only by supplying organic substrates but also by releasing redox-active metabolites and photoactive compounds that support extracellular ROS generation, reprogram bacterial metabolism, and alter local redox processes. Thus, the unresolved question is not whether algal–bacterial interfaces support metabolite and redox-active exchange, but whether such photosynthesis-driven phycosphere interactions can systematically enhance bacterial extracellular O_2_^•−^ production and thereby alter biogeochemical reactivity in the phycosphere.

Here, we used a model coculture of the bloom-forming cyanobacterium *Microcystis aeruginosa* and *Pseudomonas* sp. QJX-1 to investigate how algal–bacterial interactions regulate extracellular O_2_^•−^ production in the phycosphere. We demonstrate that photosynthesis-associated extracellular redox activity, metabolic reprogramming, and extracellular redox-active components jointly enhance bacterial extracellular O_2_^•−^ generation. By linking these microscale redox interactions to accelerated pollutant transformation and elemental cycling, this study highlights a role for biogenic ROS in shaping the biogeochemical reactivity of aquatic phycosphere microenvironments.

## Materials and methods

### Bacterial and algal strains and culture preparation

*Pseudomonas* sp. QJX-1 (GenBank accession number KM242057), a Gram-negative rod-shaped bacterium, was used as the extracellular O_2_^•−^-producing bacterial strain in this study. This strain has been deposited in the China General Microorganism Culture Center (CGMCC; accession number 6630). Unless otherwise stated, QJX-1 cells were pre-cultured in PYG medium at 30°C and 170 rpm for approximately 24 h and harvested during the logarithmic growth phase. Bacterial cells were harvested from the PYG medium by centrifugation and washed three times with sterile BG-11 medium by centrifugation (8000 rpm, 5 min; 3 cycles of centrifugation and supernatant removal).

The cyanobacterium *M. aeruginosa* was used in this study due to its prevalence in algal blooms. *Microcystis aeruginosa* (strain FACHB-905) was obtained from the Wuhan Institute of Hydrobiology, Chinese Academy of Sciences. Unless otherwise stated, *M. aeruginosa* was cultivated in BG-11 medium to the exponential growth phase, approximately 14 days, before use and washed three times with sterile BG-11 medium by centrifugation (4000 rpm, 10 min).

### Cell density measurement

The cell densities of QJX-1 and *M. aeruginosa* were determined using a NovoCyte flow cytometer (NovoCyte 1040, ACEA Biosciences, USA), and data were analyzed with NovoExpress software. Prior to analysis, culture samples were gently mixed and, when necessary, diluted with sterile BG-11 medium. To reduce persistent algal–bacterial aggregates, samples were subjected to mild dispersion by repeated pipetting followed by water-bath sonication for 30 s at 50 kHz. Background particles and debris were excluded based on forward scatter (FSC) and side scatter (SSC) signals. QJX-1 and *M. aeruginosa* were distinguished according to their scatter characteristics and the autofluorescence signal of *M. aeruginosa*, with gates defined using the corresponding monoculture controls and then applied to coculture samples. Bacterial and algal cell densities were quantified separately to monitor changes in population abundance during coculture.

### Extracellular O_2_^•−^ detection

Extracellular O_2_^•−^ levels were quantified using the superoxide-specific chemiluminescent (CL) probe MCLA (TCI, Japan) [36] in conjunction with a SPARK 10M microplate reader (Tecan, Switzerland). For each measurement, 190 μl of sample suspension was transferred into a 96-well white plate. MCLA was added to a final concentration of 3.125 μM, and the CL emission was recorded with an acquisition time of 1 s per well. Extracellular O_2_^•−^ levels were expressed as chemiluminescence unit (CLU).

To ensure the signal was derived specifically from O_2_^•−^, superoxide dismutase (SOD) (LABLEAD, China; final concentration: 1200 kU/l) was added to each well to scavenge extracellular O_2_^•−^. The specificity of the assay was confirmed by the rapid and near-complete quenching (>95%) of the CL signal upon SOD addition. The O_2_^•−^ specific signal intensity was determined by the SOD-subtraction method, calculated as the difference between the CL signal before SOD addition (CL_total_) and the residual CL signal after SOD addition (CL_background_).

The analytical sensitivity of the MCLA-based assay was evaluated to ensure reliable detection of changes in extracellular O_2_^•−^-specific chemiluminescence across different experimental treatments. The sensitivity was characterized by a signal-to-noise ratio (SNR) > 10, ensuring that all reported biological signals were clearly above the instrumental and matrix background. This sensitivity allowed reliable comparison of extracellular O_2_^•−^-specific CL signals among treatments.

## Results and discussion

### Algal-bacterial coculture enhances extracellular O_2_^•−^ Production

**Figure 1:**
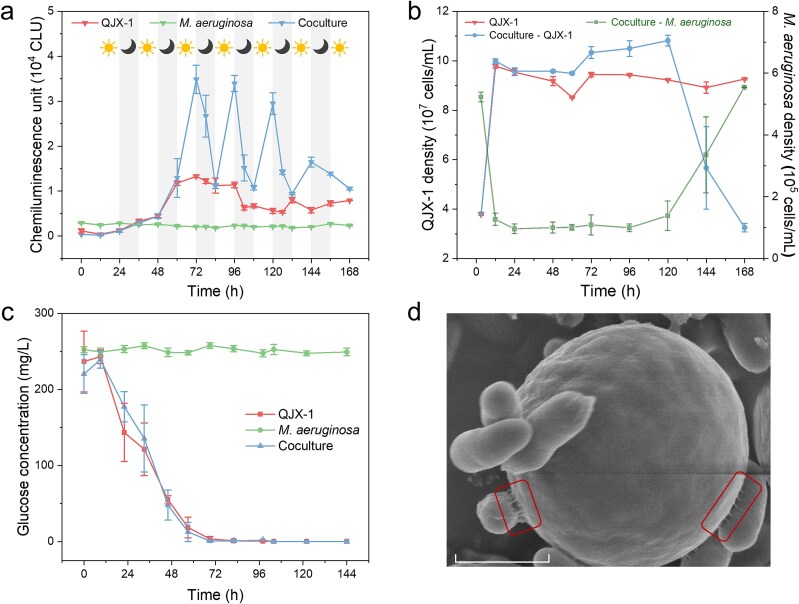
Algal–bacterial coculture enhances extracellular O_2_^•−^ production in a light-dependent manner. (a) Time-course of extracellular O_2_^•−^ production in the QJX-1 monoculture, *M. aeruginosa* monoculture, and *M. aeruginosa*–QJX-1 coculture under alternating light–dark conditions. (b) Changes in planktonic QJX-1 cell density in monoculture and coculture, and *M. aeruginosa* cell density in coculture over time. (c) Glucose consumption in the monoculture and coculture. (d) Representative scanning electron microscopy (SEM) image showing bacterial association with algal cells in coculture at 72 h. Scale bars = 1 μm. Boxes indicate representative surface-associated bacterial cells. Data are shown as mean ± s.d. from three independent biological replicates.

Cocultures of *M. aeruginosa* and QJX-1 enhanced extracellular O_2_^•−^ production. Following initial glucose depletion, O_2_^•−^ levels rose sharply in both the coculture and QJX-1 monoculture, whereas they remained at basal levels in the algal monoculture (Fig. 1a and c). The coculture exhibited a three-fold higher peak O_2_^•−^ signal and pronounced light–dark oscillations absent in the bacterial monoculture (Fig. 1a). Thus, interactions with *M. aeruginosa* were associated with a distinct, light-responsive rhythm on extracellular O_2_^•−^ accumulation.

To identify the biological source of extracellular O_2_^•−^, we employed an H-type dual-chamber setup separated by a semi-permeable membrane. O_2_^•−^ remained undetectable in the algal chamber, whereas the bacterial chamber maintained O_2_^•−^ production mirroring the coculture’s temporal pattern, albeit at reduced intensity (Fig. S1). This identifies QJX-1 as the primary O_2_^•−^ producer and reveals that although diffusible algal factors stimulate this process, direct physical proximity may be required for maximal amplification.

Glucose tracking revealed near-complete exhaustion of the initial carbon source within 48 h in both the QJX-1 monoculture and the coculture (Fig. 1c), coinciding with the onset of O_2_^•−^ accumulation (Fig. 1a). This temporal synchrony suggests that carbon starvation is an important condition promoting extracellular O_2_^•−^ production. In the QJX-1 monoculture, however, O_2_^•−^ levels declined sharply after Day 4. This likely reflects progressive metabolic exhaustion during prolonged starvation, with reduced endogenous NADH availability and lower activity of membrane-associated oxidoreductases [9, 37, 38]. Consistent with this interpretation, prolonged monoculture incubation (144 h) induced severe cellular wrinkling, deformation, and collapse—features consistent with starvation and oxidative stress [39, 40]. Conversely, bacteria physically associated with algal cells in the coculture remained morphologically intact (Figs S2 and S3), suggesting that the phycosphere may help buffer starvation-associated redox stress. Accordingly, the coculture exhibited enhanced antioxidant capacity, characterized by elevated SOD/CAT activities and higher GSH levels (Fig. S4). Furthermore, the late-stage decline in measured bulk O_2_^•−^ in the coculture may partially stem from signal quenching within surface-attached aggregates (Figs 1d, S2, and S3b), given the high reactivity and short lifetime of O_2_^•−^ within organic- and EPS-rich matrices [41, 42].

Population dynamics revealed a distinct spatial shift within the coculture. Initially, rapid bacterial proliferation coincided with reduced algal growth, possibly through competition for space and nutrients. Following carbon depletion, flow cytometry indicated a sharp decline in planktonic bacteria, whereas the cyanobacterial population rebounded (Fig. 1b). However, SEM revealed extensive bacterial colonization on algal surfaces (Figs 1d, S2, and S3), indicating a transition to a surface-associated lifestyle rather than a loss of bacterial abundance. Because colonization began prior to severe starvation and intensified over time (Fig. S2), this behavior aligns with inherent heterotrophic chemotaxis to the metabolite-rich phycosphere [43–45] rather than a strict starvation response. Furthermore, filamentous surface appendages (Fig. 1d) provide a possible structural basis for anchoring bacteria to the host, potentially facilitating their persistence in this dynamic microscale redox niche [43, 46].

### Photosynthesis increases extracellular redox activity and the accumulation of reducing equivalents in coculture

**Figure 2:**
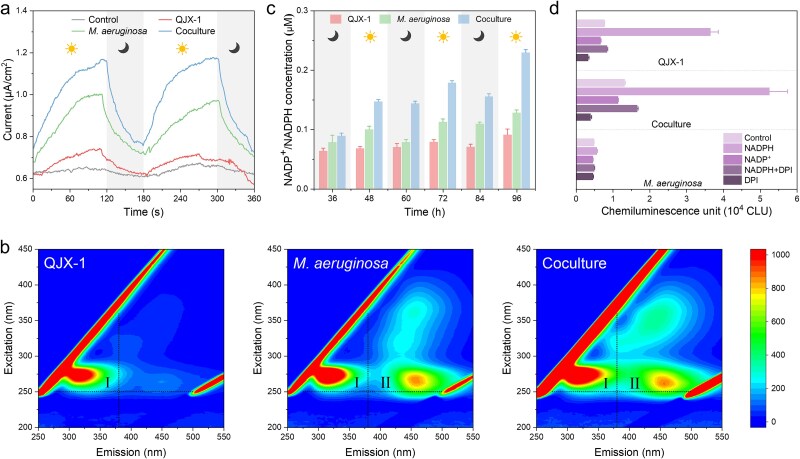
Light-responsive extracellular redox activity and total extracellular NADP(H) accumulation in the algal–bacterial coculture. (a) Photocurrent responses of the abiotic control, QJX-1 monoculture, *M. aeruginosa* monoculture, and coculture under alternating light–dark conditions. (b) Three-dimensional excitation-emission matrix fluorescence spectra of culture supernatants from the QJX-1 monoculture, *M. aeruginosa* monoculture, and coculture collected during the carbon-starvation phase. Regions I and II indicate representative fluorescence signals assigned to soluble microbial byproducts and humic/fulvic-like components, respectively. (c) Total extracellular NADP(H) concentrations in the monoculture and coculture under alternating light–dark conditions. (d) Effects of exogenous NADPH, NADP^+^, and NADPH plus DPI on extracellular O_2_^•−^ production in QJX-1 monoculture, *M. aeruginosa* monoculture, and coculture under carbon-starvation conditions. Data are shown as mean ± s.d. from three independent biological replicates.

Building upon the finding that diffusible, light-dependent algal factors stimulate bacterial O_2_^•−^ generation, we investigated the chemical nature of this extracellular redox interaction. To determine whether the coculture microenvironment undergoes broader shifts in redox reactivity, we measured light-responsive electrochemical signals. Under light–dark cycling, cocultures generated a robust photocurrent response (~1.15 μA cm^−2^), approximately 20.4% higher than *M. aeruginosa* monocultures, whereas QJX-1 monocultures exhibited negligible responses (Fig. 2a). These photocurrents indicate the extracellular transfer of reducing equivalents (e.g. NADP(H)) and redox-active metabolites by illuminated phototrophs [27], confirming that the algal–bacterial association cultivates a highly active redox microenvironment.

EEM fluorescence spectroscopy revealed a restructured extracellular DOM pool in the coculture (Fig. 2b). Specifically, the enrichment of soluble microbial byproducts (Ex/Em = 260/450 nm) is consistent with a metabolite-rich phycosphere supporting heterotrophic activity [47]. Concurrently, the increased fluorescence around Ex/Em = 350/450 nm suggests enrichment of humic/fulvic-like, redox-active DOM, indicating a coculture extracellular environment with greater potential for redox mediation [48].

Prompted by this enhanced redox-active DOM signal and the potential role of extracellular nicotinamide cofactors in microbial redox processes [31, 49], we next quantified extracellular NADP(H) using an independent enzymatic cycling assay. Levels were consistently higher under illumination and elevated in the coculture relative to monocultures (Fig. 2c). Together with the negligible photocurrent in bacterial monocultures, the light-dependent accumulation of extracellular NADP(H) supports a photosynthesis-associated enhancement of the extracellular reducing environment in coculture, alongside the observed enrichment of redox-active DOM [28, 30–32].

We next tested whether reduced nicotinamide cofactors could functionally stimulate extracellular O_2_^•−^ generation. Exogenous NADPH and NADH rapidly enhanced O_2_^•−^ production in QJX-1-containing cultures under carbon-starvation conditions, whereas the oxidized cofactors NADP^+^ and NAD^+^ had little effect (Figs 2d and S5). This stimulation was markedly attenuated by diphenyleneiodonium (DPI), an inhibitor of flavoprotein-dependent electron transfer, supporting the involvement of a bacterial NADPH oxidase (NOX)-like, NAD(P)H-responsive oxidoreductase activity in extracellular O_2_^•−^ formation. The accompanying increase in extracellular NOX-like activity in the coculture is consistent with this interpretation (Fig. S7), indicating that NAD(P)H-responsive extracellular redox activity was enhanced during the algal–bacterial interaction.

This NAD(P)H-responsive bacterial redox response underpins the observed temporal dynamics (Fig. 1a and c). Exogenous NADPH minimally stimulated O_2_^•−^ production during the carbon-replete phase, yet triggered a robust response following carbon depletion in both whole-cell suspensions and cell-free filtrates (Fig. S6). These results suggest that carbon starvation increases the capacity of QJX-1-associated extracellular redox processes to utilize reduced nicotinamide cofactors for the one-electron reduction of molecular oxygen to extracellular O_2_^•−^ [6]. Together, these findings support a microscale mechanism in which illuminated *M. aeruginosa*-containing cocultures establish a photosynthesis-associated extracellular reducing environment, while starvation-enhanced bacterial oxidoreductase-associated activity converts part of this reducing potential into localized extracellular ROS production.

### Coculture-associated siderophores promote bacterial extracellular O_2_^•−^ production

**Figure 3:**
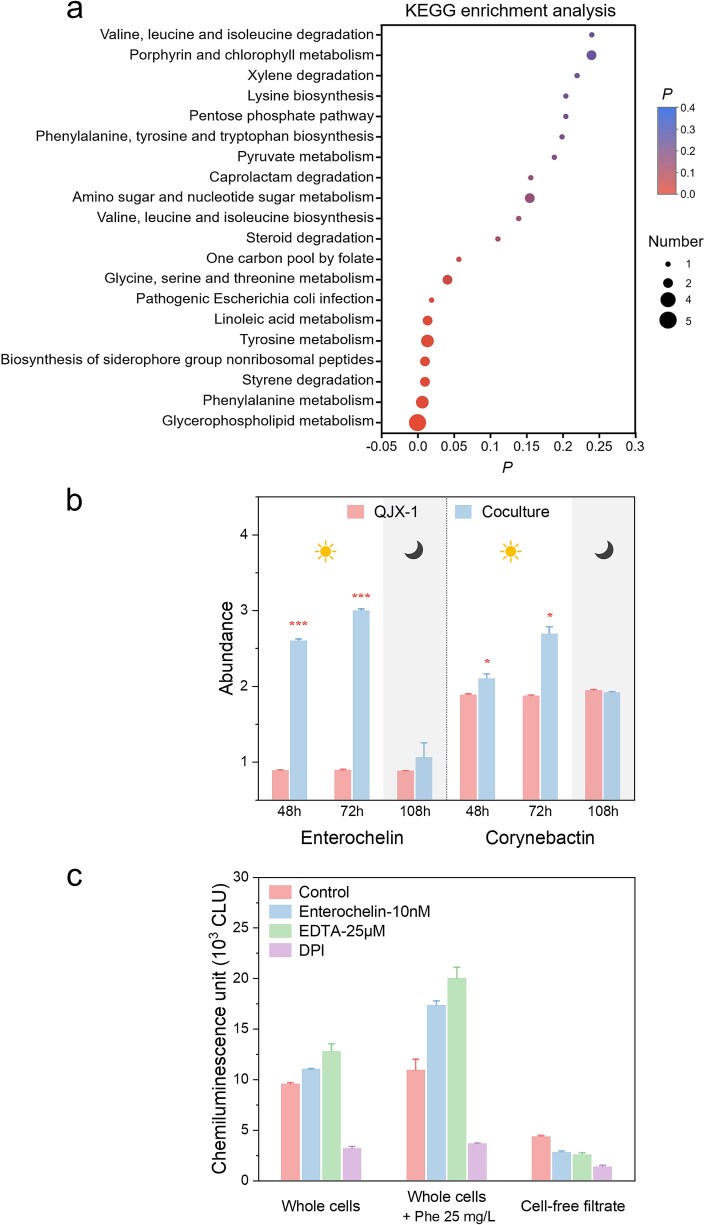
Coculture-associated siderophores promote extracellular O_2_^•−^ production by QJX-1. (a) KEGG enrichment analysis of extracellular metabolites in the *M. aeruginosa*–QJX-1 coculture relative to the QJX-1 monoculture at 72 h. (b) Relative abundances of Enterochelin and Corynebactin in the supernatants of the QJX-1 monoculture and coculture at 48, 72, and 108 h under alternating light–dark conditions. (c) Extracellular O_2_^•−^ production by QJX-1 in whole-cell suspensions, whole-cell suspensions supplemented with phenylalanine (Phe, 25 mg l^−1^), and cell-free filtrates following the addition of Enterobactin or EDTA. DPI was used as an inhibitor control. Asterisks indicate significant differences (^*^P < .05, ^**^P < .01, ^***^P < .001) compared with the corresponding control. Data are shown as mean ± s.d. from three independent biological replicates.

To further characterize the extracellular metabolites associated with the restructured DOM pool, untargeted metabolomics revealed that the coculture significantly enriched pathways for aromatic amino acid metabolism and siderophore biosynthesis (Fig. 3a). Consistent with the enrichment of soluble microbial byproducts in the EEM analysis, elevated phenylalanine, tyrosine, and related aromatic metabolites suggest an increased availability of labile aromatic substrates that may support heterotrophic activity in the phycosphere [50]. Similar to the dynamics of O_2_^•−^ production and extracellular NADP(H) accumulation, these enriched pathways were highly responsive to light–dark transitions, supporting photosynthesis-associated modulation of extracellular metabolite composition (Fig. S8, Table S1).

Specifically, two catecholate-type siderophores, Enterobactin [51, 52] and Corynebactin [53], were consistently enriched in the coculture (Fig. 3b). The chrome azurol S assay corroborated this metabolomic pattern [54], showing substantially higher siderophore levels in cocultures with pronounced light-phase peaks (Fig. S9). Because catecholate siderophore biosynthesis relies on chorismate-derived aromatic precursors (Fig. S10) [55], the concurrent enrichment of aromatic metabolites and catecholate siderophores is consistent with a link between photosynthesis-associated metabolite supply and siderophore biosynthesis [56]. Given that Fe is fiercely contested at the algal–bacterial interface, this siderophore enrichment suggests intensified microscale Fe-acquisition pressure in the coculture [56–59].

To determine whether siderophore-associated Fe perturbation could enhance cell-associated O_2_^•−^ production, we exposed QJX-1 cultures to Enterobactin or EDTA. Both strong chelators stimulated extracellular O_2_^•−^ generation over a defined concentration range (Fig. S11). Whole-cell suspensions exhibited robust O_2_^•−^ production, whereas cell-free filtrates showed little to no response (Fig. 3c). These results indicate that siderophore-enhanced O_2_^•−^ production was primarily associated with metabolically active bacterial cells and was not explained by chelator-driven extracellular chemistry alone. Furthermore, the magnitude of this response was tightly linked to the starvation switch. Stimulation was negligible during the carbon-replete phase, peaked during early carbon starvation, and waned during prolonged starvation (Fig. S12). Replenishing late-starved cells with glucose or representative algal-derived aromatic amino acids restored the siderophore-induced O_2_^•−^ response (Figs S13–S15). These dynamics suggest that siderophore-enhanced O_2_^•−^ production requires metabolically active cells with sufficient upstream electron supply, supporting a role for algal-derived metabolites in sustaining bacterial redox responsiveness under carbon-starvation conditions. Comparable responses across multiple bacterial strains suggest that this siderophore/chelator-associated stimulation reflects a broader bacterial physiological response rather than a QJX-1-specific trait.

To further examine whether this cell-associated response was linked to bacterial respiratory redox processes, we investigated the impact of strong Fe-chelators on respiratory-related components. Spectroscopic analysis of purified cytochrome *c* incubated with Enterobactin revealed a reduced Soret band intensity and a blueshift from 409 to 405 nm (Fig. S16), suggesting that catecholate siderophores can alter the heme microenvironment of Fe-containing redox proteins under defined conditions [60]. Furthermore, inhibiting the respiratory electron transport chain (ETC) with rotenone severely attenuated the Enterobactin-induced O_2_^•−^ production (Fig. S17). Together, these data suggest that siderophore/chelator-associated Fe perturbation can alter bacterial respiratory redox balance, thereby contributing to enhanced extracellular O_2_^•−^ formation [61, 62]. Consistent with the involvement of electron-partitioning processes, supplementing the system with NaNO_3_ as an alternative electron sink consistently decreased extracellular O_2_^•−^ (Fig. S18a) without affecting biomass or dissolved O_2_ levels (Fig. S18b). These results suggest that redirecting reducing equivalents toward an alternative electron acceptor can attenuate extracellular O_2_^•−^ production, further supporting a respiratory redox contribution to this response.

Together, these results suggest that algal-derived metabolites help sustain bacterial redox responsiveness under carbon-starvation conditions, while siderophore/chelator-associated Fe perturbation modulates respiratory redox balance and thereby contributes to enhanced extracellular O_2_^•−^ production at the algal–bacterial interface.

### Transcriptomic evidence supports metabolic and respiratory reprogramming in coculture

To validate these mechanisms at the molecular level, we profiled the coculture transcriptomes during carbon depletion and starvation. Principal component analysis revealed clear clustering driven by growth stage and culture condition, indicating that nutrient status and algal–bacterial interactions systematically reshape global transcription (Fig. S19).

In QJX-1, carbon starvation extensively remodeled central carbon metabolism. The systemic downregulation of major TCA cycle enzymes, coupled with the upregulation of isocitrate lyase (the key glyoxylate shunt enzyme), indicates a shift toward carbon conservation (Fig. S20). This metabolic pivot restricts the endogenous electron donor supply [63–65], providing a transcriptional basis for the eventual decline in extracellular O_2_^•−^ production during prolonged starvation (Fig. 1a). Concurrently, the robust induction of genes encoding amino acid transport, aromatic amino acid utilization, and ABC transport systems (Fig. S21) aligns with the metabolomic enrichment of algal-derived aromatic subsidies. This transcriptional signature confirms that the coculture provisions the organic substrates essential for sustaining bacterial basal metabolism—and consequently, respiratory redox activity—throughout the starvation phase.

**Figure 4:**
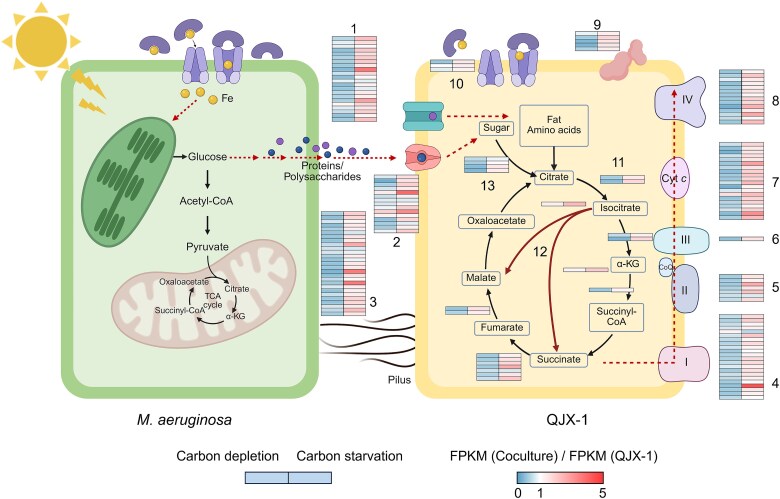
Coculture induces coordinated metabolic, respiratory and Fe-acquisition reprogramming during extracellular O_2_^•−^ production. Schematic summary of transcriptomic responses in the *M. aeruginosa*-QJX-1 coculture during the carbon-depletion and carbon-starvation stages. Heat maps indicate the relative transcript abundance of genes associated with key physiological processes, including amino acid transport (1), sugar transport (2), pilus biogenesis (3), NADH dehydrogenase (4), succinate dehydrogenase (5), cytochrome *c* reductase (6), cytochrome-associated functions (7), cytochrome *c* oxidase (8), catalase (9), siderophore-mediated Fe transport (10), the TCA cycle (11), the glyoxylate shunt (12), and pyruvate dehydrogenase (13). Heat map values are expressed as the ratio of FPKM values in coculture to those in the QJX-1 monoculture. A value of 1 indicates no change; values >1 indicate higher expression in coculture, whereas values <1 indicate lower expression relative to the QJX-1 monoculture. For each heat map, the left column represents the carbon-depletion stage and the right column represents the carbon-starvation stage. Additional gene annotations are provided in Table S2. Image created with BioRender.com.

Parallel to this metabolic adaptation, the coculture induced highly coordinated transcriptomic responses within the bacterial ETC. Upregulation of genes governing NADH dehydrogenase activity, cytochrome assembly, and Fe-S cluster biogenesis (Figs 4 and S22) indicates a profound restructuring of Fe-dependent respiration. Rather than increasing respiratory capacity, this likely reflects compensatory maintenance of Fe-containing machinery [66, 67]. We hypothesize this respiratory remodeling stems from siderophore-associated Fe competition and microenvironmental Fe deficiency. Although in vitro data show strong siderophores can alter the heme microenvironment of model Fe-proteins (Figs S11 and S16), prevailing Fe scarcity restricts their resynthesis, contributing to a persistent respiratory redox imbalance [68, 69].

To manage this altered respiratory redox flow, the concurrent upregulation of alternative electron acceptor pathways (e.g. nitrate reduction genes *nap* and *nirB/D*) reflects an active metabolic strategy to safely reroute excess reducing power (Table S2). Concurrently, *M. aeruginosa* upregulated its own Fe-acquisition genes, including Fe-siderophore ABC transporter permeases (Fig. S23). This signature reveals active algal siderophore piracy, utilizing bacterially excreted siderophores to fulfill algal Fe demands [70, 71]. This asymmetric competition intensifies Fe scarcity, driving compensatory bacterial siderophore overproduction. Consequently, competitive Fe-scavenging traps bacteria in chronic Fe-stress and respiratory perturbation, predisposing the respiratory chain to incomplete oxygen reduction and O_2_^•−^ formation. Despite the resulting oxidative burden, upregulated antioxidant defenses successfully mitigate this stress (Figs S2 and S4). Ultimately, this respiratory inefficiency represents a necessary ecological trade-off: bacteria tolerate siderophore-associated respiratory redox imbalance to secure essential carbon subsidies from the phycosphere.

Providing a molecular basis for the physical algal–bacterial associations (Fig. 1d), we identified a marked upregulation of genes governing pilus biogenesis, assembly, and twitching motility during carbon starvation in the coculture (Table S2). Consistent with our morphological observations, the active induction of these appendages provides the genetic and structural basis for transitioning to a surface-attached lifestyle [46, 72], thereby establishing a tightly coupled spatial architecture [73]. This physical proximity is critical for maximizing metabolite exchange, facilitating localized Fe competition, and sustaining the microscale interactions driving O_2_^•−^ generation.

Collectively, our integrated transcriptomic, metabolomic, and physiological data support a model in which algal carbon provisioning and siderophore- associated respiratory remodeling synergistically contribute to bacterial respiratory redox imbalance, establishing the phycosphere as a prominent microenvironmental hotspot for extracellular ROS generation.

### Extracellular redox-active and photoactive components broaden the ecological relevance of algal–bacterial ROS production

**Figure 5:**
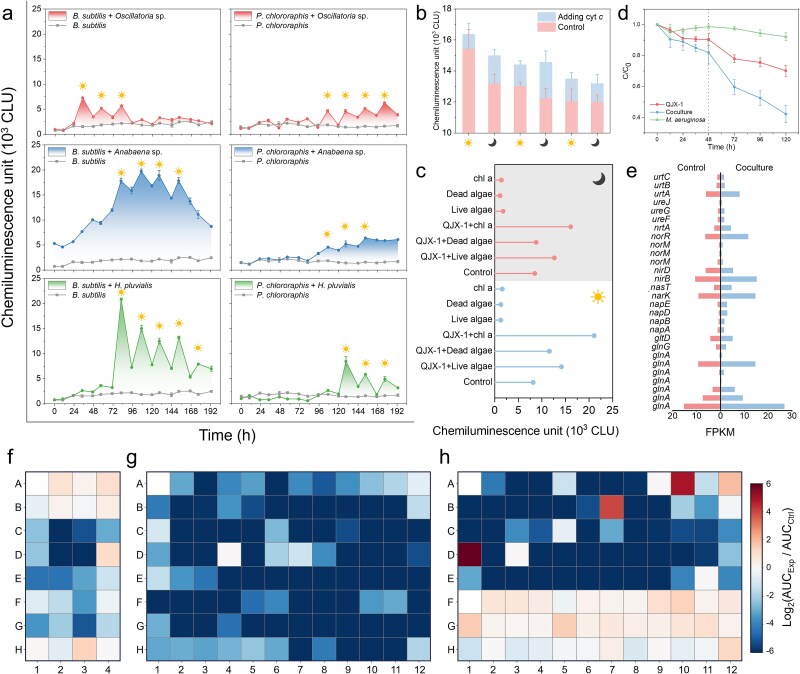
Extracellular ROS link redox interactions with pollutant transformation and elemental metabolism in algal–bacterial coculture. (a) Extracellular O₂^•−^ production by heterotrophic bacteria cocultured with different algal species under alternating light–dark conditions. The initial inoculum densities and culture conditions were consistent with the previous experiments. (b) Effects of exogenous cytochrome *c* on extracellular O₂^•−^ production by QJX-1 under light and dark conditions. (c) Effects of heat-killed *M. aeruginosa* cells and purified chlorophyll *a* on extracellular O₂^•−^ production by QJX-1. Heat-killed algal cells were prepared by incubating *M. aeruginosa* at 65°C for 30 min, and the concentration of chlorophyll *a* was equivalent to that extracted from the same algal cell density. (d) Removal of sulfamethoxazole (SMX) in the *M. aeruginosa* monoculture, QJX-1 monoculture, and coculture. C/C_0_ indicates the residual fraction of SMX relative to the initial concentration (initial SMX concentration, 50 μg l^−1^). (e) Relative transcript abundance of nitrogen cycle-related genes during the carbon-starvation stage in coculture relative to the QJX-1 monoculture. Additional gene annotations are provided in Table S3. (f)-(h) Heat maps showing the effects of extracellular ROS quenching on substrate utilization in the algal–bacterial coculture, including carbon sources (f; ECO plate), nitrogen sources (g; PM3 plate), and phosphorus and sulfur sources (h; PM4 plate). In (h), rows A–E represent phosphorus sources and rows F–H represent sulfur sources. Heat map values indicate log_2_ fold changes in AUC in the ROS-quenching treatment relative to the untreated starved coculture. Negative values indicate reduced metabolic activity after ROS quenching. Additional substrate information is provided in Table S4. Data are shown as mean ± s.d. from three independent biological replicates where applicable.

Building on the observed respiratory responses, we investigated whether extracellular redox-active or photoactive components could further enhance this bacterial O_2_^•−^ production. Aligning with the upregulated cytochrome-associated functions (Fig. S22b), exogenous cytochrome *c* significantly stimulated O_2_^•−^ production in QJX-1. However, this light-independent effect was attenuated during prolonged starvation (Fig. 5b), indicating that this enhancement is tightly linked to bacterial metabolic state rather than representing a purely abiotic extracellular reaction [74]. Furthermore, given the light-driven nature of coculture O_2_^•−^ generation, we evaluated representative algal photoactive components [35]. Neither heat-killed *M. aeruginosa* cells nor purified chlorophyll *a* alone generated substantial O_2_^•−^, but both enhanced illuminated QJX-1 O_2_^•−^ production, with chlorophyll *a* exhibiting the strongest effect (Fig. 5c). The comparatively weaker response from dead biomass likely reflects simultaneous ROS quenching by bulk organics or steric hindrance. Collectively, these results indicate that beyond exchanging dissolved metabolites, released photosynthetic pigments and extracellular redox-active compounds can further enhance localized bacterial O_2_^•−^ production under specific conditions, although their proportional contributions in situ remain to be quantified.

This photosynthesis-associated enhancement of extracellular O_2_^•−^ is not unique to the *M. aeruginosa*-QJX-1 pairing. Across diverse algal–bacterial combinations, cocultures consistently exhibited higher extracellular O_2_^•−^ production than monocultures and maintained a light-dependent rhythm (Fig. 5a). Despite varying magnitudes of enhancement, this pattern indicates that photosynthesis-driven induction of extracellular O_2_^•−^ is a widespread physiological response. Ecologically, these interactions likely establish dynamic, microscale zones of elevated redox reactivity across sunlit surface waters.

This sustained biogenic O_2_^•−^ production confers distinct environmental and physiological advantages. Using sulfamethoxazole (SMX) as a model micro-pollutant, the coculture achieved significantly higher removal (57.8% after 120 h) than either the QJX-1 (29.9%) or *M. aeruginosa* (7.9%) monocultures (Fig. 5d). Segmented pseudo-first-order kinetic analysis revealed a key temporal correlation: although initial degradation rates (0–48 h) were comparable, the onset of carbon starvation (48–120 h) accelerated degradation in the coculture (*k* = 0.0100 h^−1^), whereas the monoculture rate plateaued (*k* = 0.0038 h^−1^) (Fig. S24). This acceleration coincides with peak extracellular O_2_^•−^ production (Fig. 1a), indicating that starvation- enhanced biogenic ROS contributes to the pre-oxidative transformation of recalcitrant pollutants. Furthermore, phenotypic microarrays demonstrated that quenching extracellular ROS broadly impaired coculture metabolic activity under diverse chemical stressors (antibiotics, fungicides, heavy metals) (Fig. S25). This indicates extracellular ROS may serve as a critical chemical defense, contributing to broad-spectrum stress tolerance within the micro-consortium [3].

Beyond defensive functions, extracellular ROS is linked to elemental cycling and metabolic plasticity. During carbon starvation (coinciding with maximal O_2_^•−^ output), genes governing organic nitrogen utilization, ammonium assimilation, and dissimilatory nitrate reduction were coordinately upregulated (Fig. 5e). This reorganization extends beyond nutritional scavenging; as noted above, nitrate reduction may act as a compensatory electron sink, coupling elemental nitrogen turnover with respiratory ROS dynamics. Functionally, quenching extracellular ROS severely suppressed the utilization of most nitrogen substrates, particularly amino acids and amines (Fig. 5g). Parallel suppression occurred across diverse carbon and phosphorus sources, primarily impairing the breakdown of complex polyols, carboxylates, and hydroxy-aromatics (Fig. 5f and h). Conversely, sulfur metabolism was the sole exception: utilization of sulfur substrates linked to redox buffering (e.g. glutathione precursors) slightly increased following ROS quenching (Fig. 5h). This implies a potential rapid, compensatory upregulation of sulfur-based antioxidant pathways when the native extracellular redox regime is disrupted. Collectively, these functional responses suggest the consortium’s capacity to actively rewire elemental networks to maintain systemic redox homeostasis.

### A working model for photosynthesis-enhanced extracellular O_2_^•−^ production in algal–bacterial systems

**Figure 6:**
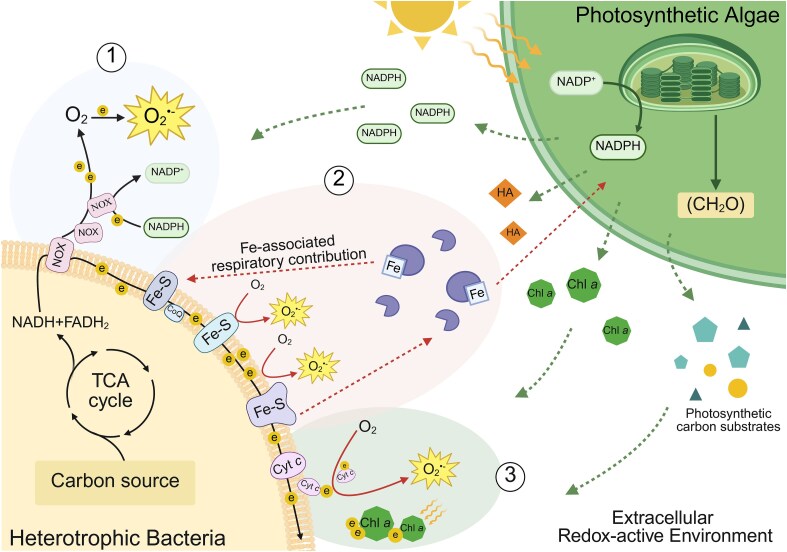
Proposed working model for photosynthesis-enhanced bacterial extracellular O_2_^•−^ production in the algal–bacterial phycosphere. The schematic summarizes interacting phycosphere-associated processes that contribute to QJX-1-associated extracellular O_2_^•−^ production at the algal–bacterial interface. (1) NAD(P)H-responsive pathway: Illuminated cocultures establish a photosynthesis-associated extracellular redox-active environment. Algal-derived reduced nicotinamide cofactors (NADPH) stimulate a diphenyleneiodonium (DPI)-sensitive, bacterial NOX-like oxidoreductase activity, driving the one-electron reduction of molecular oxygen to extracellular O_2_^•−^. This activity is localized at the bacterial cell surface/interface, consistent with the detected membrane-associated and extracellular NOX-like activity. This model serves as a functional descriptor and does not assign this activity to a specific molecularly identified enzyme. (2) Siderophore-associated pathway: During carbon starvation, algal metabolites sustain bacterial redox responsiveness, while microscale Fe competition and catecholate siderophore accumulation drive respiratory redox modulation, acting as a respiration-linked regulatory contribution to enhanced bacterial O_2_^•−^ formation. (3) Additional environmental enhancement: Representative extracellular redox-active and algal photoactive components (e.g. cytochrome *c* and chlorophyll *a*) further modulate and enhance the bacterial O_2_^•−^ response under defined experimental conditions. Created with BioRender.com.

Synthesizing our multi-omics and physiological data, we propose a comprehensive working model for extracellular O_2_^•−^ production at the algal–bacterial interface (Fig. 6). This enhanced biogenic ROS production is associated with three interacting phycosphere processes. First, illuminated algal-containing cocultures establish a photosynthesis-associated extracellular redox-active environment, as supported by enhanced photocurrent, redox-active DOM enrichment, and elevated extracellular NADP(H). Reduced nicotinamide cofactors can stimulate DPI-sensitive bacterial oxidoreductase-associated processes that promote the one-electron reduction of molecular oxygen to extracellular O_2_^•−^. Second, during carbon starvation, algal-derived metabolites can help sustain bacterial redox responsiveness, while coculture-associated catecholate siderophore accumulation is consistent with intensified microscale Fe competition. Siderophore-associated Fe perturbation may modulate bacterial respiratory redox balance, thereby contributing to enhanced extracellular O_2_^•−^ formation. Third, representative extracellular redox-active and algal photoactive components further modulate bacterial O_2_^•−^ production under defined conditions. Cytochrome *c* stimulated bacterial O_2_^•−^ production, whereas chlorophyll *a* enhanced O_2_^•−^ production in illuminated bacterial suspensions but generated little O_2_^•−^ alone.

Although presented separately for clarity, these processes are best viewed as interacting contributors rather than independent additive pathways. The NAD(P)H-responsive bacterial redox process provides the most direct functional link to extracellular O_2_^•−^ formation, whereas the siderophore-associated process represents a respiration-linked regulatory contribution. Redox-active and photoactive components appear to provide additional enhancement under defined conditions. The relative importance of these contributors is likely context-dependent: siderophore-associated respiratory modulation may become more prominent under stronger Fe limitation, whereas chlorophyll-containing photoactive materials may have greater influence during algal senescence or bloom decay. Structurally, the close spatial organization of the phycosphere may reinforce these processes by increasing local exposure to diffusible algal products, redox-active organic matter, siderophores, and algal photoactive components. Together, photosynthesis, carbon limitation, Fe competition, and extracellular redox-active components shape a dynamic phycosphere microenvironment that favors bacterial extracellular superoxide production.

## Conclusion

This study demonstrates that photosynthetic algae enhance extracellular O_2_^•−^ production by heterotrophic bacteria, establishing the phycosphere as a dynamic microscale redox environment. Across diverse algal–bacterial pairings, enhanced extracellular O_2_^•−^ production is closely associated with illumination, accelerated by carbon depletion, and stimulated by the availability of reducing equivalents and siderophores. Consequently, photosynthesis extends beyond carbon provisioning to modulate extracellular redox chemistry, facilitating ROS generation. This phenomenon is likely widespread in oligotrophic, Fe-limited, and light-fluctuating natural waters, where continuous biogenic ROS generation contributes to organic matter transformation, trace metal cycling, and microscale elemental bioavailability.

Biogeochemically, this ROS-producing phenotype carries critical environmental implications. The high-ROS coculture microenvironment was associated with accelerated oxidative transformation of recalcitrant pollutants (e.g. sulfamethoxazole), enhanced community-level tolerance to broad-spectrum chemical stressors, and coordinated shifts in elemental metabolism. Thus, rather than mere reactive byproducts, biogenic ROS can function as important factors linking contaminant degradation with redox-dependent nutrient turnover and stress resilience in photic-zone microbiomes.

Beyond microscale interactions, this high-ROS phenotype is dynamically modulated by environmental conditions. Extracellular O_2_^•−^ production peaks under temperatures optimal for algal photometabolism but is rapidly suppressed by shading or transient glucose repletion (Fig. S26). This environmental gating implies that this process’s ecological impact fluctuates dynamically across seasons, depths, and trophic states. Although this study characterizes these interactions within controlled models, future investigations integrating in situ observations, community metatranscriptomics, and gradient experiments are required to quantify how photosynthesis-driven phycosphere interactions shape global aquatic ROS dynamics, natural contaminant attenuation, and biogeochemical cycling.

### Data analysis

All experiments were performed in triplicate unless otherwise noted, and results are presented as the mean values with standard errors. Figures were generated using Origin 8.5. Statistical analyses were performed using one-way analysis of variance (ANOVA), followed by the least significant difference test (*P* < .05) (SPSS Version 21.0).

## Supplementary Material

Supplementary_material_wrag175

## Data Availability

The datasets generated during and/or analyzed during the current study are available in the Zenodo repository, https://doi.org/10.5281/zenodo.20717758.

## References

[1] Hansel CM, Diaz JM. Production of extracellular reactive oxygen species by marine biota. *Ann Rev Mar Sci* 2021;13:177–200. 10.1146/annurev-marine-041320-102550 32956016

[2] Morris JJ, Rose AL, Lu Z. Reactive oxygen species in the world ocean and their impacts on marine ecosystems. *Redox Biol* 2022;52:102285. 10.1016/j.redox.2022.102285 35364435 PMC8972015

[3] Zhou S, An W, Gan C, et al. Environmental implications of bacterial-derived extracellular reactive oxygen species. *Int Biodeter Biodegr* 2024;187:105706. 10.1016/j.ibiod.2023.105706

[4] Sutherland KM, Wankel SD, Hansel CM. Dark biological superoxide production as a significant flux and sink of marine dissolved oxygen. *Proc Natl Acad Sci U S A* 2020;117:3433–9. 10.1073/pnas.1912313117 32015131 PMC7035516

[5] Sutherland KM, Coe A, Gast RJ, et al. Extracellular superoxide production by key microbes in the global ocean. *Limnol Oceanogr* 2019;64:2679–93. 10.1002/lno.11247

[6] Diaz JM, Hansel CM, Voelker BM, et al. Widespread production of extracellular superoxide by heterotrophic bacteria. *Science* 2013;340:1223–6. 10.1126/science.1237331 23641059

[7] Plummer S, Taylor AE, Harvey EL, et al. Dynamic regulation of extracellular superoxide production by the coccolithophore *Emiliania huxleyi* (CCMP 374). *Front Microbiol* 2019;10:1546. 10.3389/fmicb.2019.01546 31354655 PMC6640029

[8] Lara-Ortíz T, Riveros-Rosas H, Aguirre J. Reactive oxygen species generated by microbial NADPH oxidase NoxA regulate sexual development in *Aspergillus nidulans*. *Mol Microbiol* 2003;50:1241–55. 10.1046/j.1365-2958.2003.03800.x 14622412

[9] Hansel CM, Diaz JM, Plummer S. Tight regulation of extracellular superoxide points to its vital role in the physiology of the globally relevant *Roseobacter* clade. *mBio* 2019;10:e02668–18. 10.1128/mBio.02668-18 PMC6414704 30862752

[10] Diaz JM, Plummer S, Hansel CM, et al. NADPH-dependent extracellular superoxide production is vital to photophysiology in the marine diatom *Thalassiosira oceanica*. *Proc Natl Acad Sci U S A* 2019;116:16448–53. 10.1073/pnas.1821233116 31346083 PMC6697786

[11] Learman DR, Voelker BM, Vazquez-Rodriguez AI, et al. Formation of manganese oxides by bacterially generated superoxide. *Nat Geosci* 2011;4:95–8. 10.1038/ngeo1055

[12] Li HP, Daniel B, Creeley D, et al. Superoxide production by a manganese-oxidizing bacterium facilitates iodide oxidation. *Appl Environ Microbiol* 2014;80:2693–9. 10.1128/AEM.00400-14 24561582 PMC3993295

[13] Wang L, Ye L, Yin Z, et al. Antimonite oxidation by microbial extracellular superoxide in *Pseudomonas* sp. SbB1. *Geochim Cosmochim Acta* 2022;316:122–34. 10.1016/j.gca.2021.10.019

[14] Andeer PF, Learman DR, McIlvin M, et al. Extracellular haem peroxidases mediate Mn(II) oxidation in a marine *Roseobacter* bacterium via superoxide production. *Environ Microbiol* 2015;17:3925–36. 10.1111/1462-2920.12893 25923595

[15] Zhang X, Guo Y, Liu G, et al. Superoxide-mediated extracellular mercury reduction by aerobic marine bacterium *Alteromonas* sp. KD01. *Environ Sci Technol* 2023;57:20595–604. 10.1021/acs.est.3c04777 38007712

[16] Li Z, Wang J, Gu C, et al. Marine bacteria-mediated abiotic-biotic coupling degradation mechanism of ibuprofen. *J Hazard Mater* 2022;435:128960. 10.1016/j.jhazmat.2022.128960 35472552

[17] Zhang T, Hansel CM, Voelker BM, et al. Extensive dark biological production of reactive oxygen species in brackish and freshwater ponds. *Environ Sci Technol* 2016;50:2983–93. 10.1021/acs.est.5b03906 26854358

[18] Rose AL, Webb EA, Waite TD, et al. Measurement and implications of nonphotochemically generated superoxide in the equatorial Pacific Ocean. *Environ Sci Technol* 2008;42:2387–93. 10.1021/es7024609 18504970

[19] Bell W, Mitchell R. Chemotactic and growth responses of marine bacteria to algal extracellular products. *Biol Bull* 1972;143:265–77. 10.2307/1540052

[20] Seymour JR, Amin SA, Raina JB, et al. Zooming in on the phycosphere: the ecological interface for phytoplankton–bacteria relationships. *Nat Microbiol* 2017;2:17065. 10.1038/nmicrobiol.2017.65 28555622

[21] Mitchell JG, Okubo A, Fuhrman JA. Microzones surrounding phytoplankton form the basis for a stratified marine microbial ecosystem. *Nature* 1985;316:58–9. 10.1038/316058a0

[22] Liu F, Gledhill M, Tan QG, et al. Phycosphere pH of unicellular nano- and micro- phytoplankton cells and consequences for iron speciation. *ISME J* 2022;16:2329–36. 10.1038/s41396-022-01280-1 35798938 PMC9478132

[23] Cirri E, Pohnert G. Algae−bacteria interactions that balance the planktonic microbiome. *New Phytol* 2019;223:100–6. 10.1111/nph.15765 30825329

[24] Teeling H, Fuchs BM, Becher D, et al. Substrate-controlled succession of marine bacterioplankton populations induced by a phytoplankton bloom. *Science* 2012;336:608–11. 10.1126/science.1218344 22556258

[25] Kim H, Kimbrel JA, Vaiana CA, et al. Bacterial response to spatial gradients of algal-derived nutrients in a porous microplate. *ISME J* 2022;16:1036–45. 10.1038/s41396-021-01147-x 34789844 PMC8940921

[26] Focardi A, Bramucci AR, Ajani P, et al. Defining the ecological strategies of phytoplankton associated bacteria. *Nat Commun* 2025;16:6363. 10.1038/s41467-025-61523-5 40640136 PMC12246461

[27] Wroe EI, Egan RM, Willyam SJ, et al. Harvesting photocurrents from cyanobacteria and algae. *Curr Opin Electrochem* 2024;46:101535. 10.1016/j.coelec.2024.101535

[28] Shlosberg Y, Schuster G, Adir N. Harnessing photosynthesis to produce electricity using cyanobacteria, green algae, seaweeds and plants. *Front Plant Sci* 2022;13:955843. 10.3389/fpls.2022.955843 35968083 PMC9363842

[29] Saper G, Kallmann D, Conzuelo F, et al. Live cyanobacteria produce photocurrent and hydrogen using both the respiratory and photosynthetic systems. *Nat Commun* 2018;9:2168. 10.1038/s41467-018-04613-x 29867170 PMC5986869

[30] Shlosberg Y, Tóth TN, Eichenbaum B, et al. Electron mediation and photocurrent enhancement in *Dunalliela salina* driven bio-photo electrochemical cells. *Catalysts* 2021;11:1220. 10.3390/catal11101220

[31] Shlosberg Y, Eichenbaum B, Tóth TN, et al. NADPH performs mediated electron transfer in cyanobacterial-driven bio-photoelectrochemical cells. *iScience* 2021;24:101892. 10.1016/j.isci.2020.101892 33364581 PMC7750406

[32] Shlosberg Y, Krupnik N, Tóth TN, et al. Bioelectricity generation from live marine photosynthetic macroalgae. *Biosens Bioelectron* 2022;198:113824. 10.1016/j.bios.2021.113824 34864244

[33] Landa M, Burns AS, Roth SJ, et al. Bacterial transcriptome remodeling during sequential co-culture with a marine dinoflagellate and diatom. *ISME J* 2017;11:2677–90. 10.1038/ismej.2017.117 28731474 PMC5702724

[34] Barak-Gavish N, Dassa B, Kuhlisch C, et al. Bacterial lifestyle switch in response to algal metabolites. *eLife* 2023;12:e84400. 10.7554/eLife.84400 36691727 PMC9873259

[35] Chen S, Chen J, Zhang L, et al. Biophotoelectrochemical process co-driven by dead microalgae and live bacteria. *ISME J* 2023;17:712–9. 10.1038/s41396-023-01383-3 36823233 PMC10119253

[36] Godrant A, Rose A, Sarthou G, et al. New method for the determination of extracellular production of superoxide by marine phytoplankton using the chemiluminescence probes MCLA and red-CLA. *Limnol Oceanogr Methods* 2009;7:682–92. 10.4319/lom.2009.7.682

[37] He Z, Li Q, Xu Y, et al. Production of extracellular superoxide radical in microorganisms and its environmental implications: a review. *Environ Pollut* 2023;338:122563. 10.1016/j.envpol.2023.122563 37717891

[38] Smirnova GV, Tyulenev AV, Muzyka NG, et al. The sharp phase of respiratory inhibition during amino acid starvation in *Escherichia coli* is RelA-dependent and associated with regulation of ATP synthase activity. *Res Microbiol* 2018;169:157–65. 10.1016/j.resmic.2018.02.003 29477583

[39] Shi H, Westfall CS, Kao J, et al. Starvation induces shrinkage of the bacterial cytoplasm. *Proc Natl Acad Sci U S A* 2021;118:e2104686118. 10.1073/pnas.2104686118 34117124 PMC8214708

[40] Zhang H, Wang Z, Li Z, et al. L-glycine and l-glutamic acid protect *Pediococcus pentosaceus* R1 against oxidative damage induced by hydrogen peroxide. *Food Microbiol* 2022;101:103897. 10.1016/j.fm.2021.103897 34579850

[41] da Cruz Nizer WS, Adams ME, Allison KN, et al. Oxidative stress responses in biofilms. *Biofilm* 2024;7:100203. 10.1016/j.bioflm.2024.100203 38827632 PMC11139773

[42] Heller MI, Croot PL. Kinetics of superoxide reactions with dissolved organic matter in tropical Atlantic surface waters near Cape Verde (TENATSO). *J Geophys Res: Oceans* 2010;115:C12038. 10.1029/2009JC006021

[43] Tanabe Y, Yamaguchi H, Yoshida M, et al. Characterization of a bloom-associated alphaproteobacterial lineage, ‘*Candidatus* Phycosocius’: insights into freshwater algal-bacterial interactions. *ISME Commun* 2023;3:20. 10.1038/s43705-023-00228-6 36906708 PMC10008586

[44] Wang FQ, Bartosik D, Sidhu C, et al. Particle-attached bacteria act as gatekeepers in the decomposition of complex phytoplankton polysaccharides. *Microbiome* 2024;12:32. 10.1186/s40168-024-01757-5 38374154 PMC10877868

[45] Lipsman V, Shlakhter O, Rocha J, et al. Bacteria contribute exopolysaccharides to an algal-bacterial joint extracellular matrix. *NPJ Biofilms Microbiomes* 2024;10:36. 10.1038/s41522-024-00510-y 38561371 PMC10984933

[46] Dang H, Lovell CR. Microbial surface colonization and biofilm development in marine environments. *Microbiol Mol Biol Rev* 2015;80:91–138. 10.1128/MMBR.00037-15 26700108 PMC4711185

[47] Raina JB, Giardina M, Brumley DR, et al. Chemotaxis increases metabolic exchanges between marine picophytoplankton and heterotrophic bacteria. *Nat Microbiol* 2023;8:510–21. 10.1038/s41564-023-01327-9 36759754

[48] Yang Z, Kappler A, Jiang J. Reducing capacities and distribution of redox-active functional groups in low molecular weight fractions of humic acids. *Environ Sci Technol* 2016;50:12105–13. 10.1021/acs.est.6b02645 27759370

[49] Shlosberg Y, Spungin D, Schuster G, et al. *Trichodesmium erythraeum* produces a higher photocurrent than other cyanobacterial species in bio-photo electrochemical cells. *Biochim Biophys Acta Bioenerg* 2022;1863:148910. 10.1016/j.bbabio.2022.148910 35944660

[50] Ferrer-González FX, Widner B, Holderman NR, et al. Resource partitioning of phytoplankton metabolites that support bacterial heterotrophy. *ISME J* 2021;15:762–73. 10.1038/s41396-020-00811-y 33097854 PMC8027193

[51] Pollack JR, Neilands JB. Enterobactin, an iron transport compound from *salmonella typhimurium*. *Biochem Biophys Res Commun* 1970;38:989–92. 10.1016/0006-291X(70)90819-3 4908541

[52] Raymond KN, Dertz EA, Kim SS. Enterobactin: an archetype for microbial iron transport. *Proc Natl Acad Sci U S A* 2003;100:3584–8. 10.1073/pnas.0630018100 12655062 PMC152965

[53] Budzikiewicz H, Bössenkamp A, Taraz K, et al. Corynebactin, a cyclic catecholate siderophore from *Corynebacterium glutamicum* ATCC 14067 (*Brevibacterium* sp. DSM 20411). *Z Naturforsch C* 1997;52:551–4. 10.1515/znc-1997-7-820

[54] Louden B, Haarmann D, Lynne A. Use of blue agar CAS assay for siderophore detection. *J Microbiol Biol Educ* 2011;12:51–3. 10.1128/jmbe.v12i1.249 23653742 PMC3577196

[55] Gehring AM, Bradley KA, Walsh CT. Enterobactin biosynthesis in *Escherichia coli*: isochorismate lyase (EntB) is a bifunctional enzyme that is phosphopantetheinylated by EntD and then acylated by EntE using ATP and 2,3-dihydroxybenzoate. *Biochemistry* 1997;36:8495–503. 10.1021/bi970453p 9214294

[56] Amin SA, Green DH, Hart MC, et al. Photolysis of iron–siderophore chelates promotes bacterial–algal mutualism. *Proc Natl Acad Sci U S A* 2009;106:17071–6. 10.1073/pnas.0905512106 19805106 PMC2761308

[57] Sandy M, Butler A. Microbial iron acquisition: marine and terrestrial siderophores. *Chem Rev* 2009;109:4580–95. 10.1021/cr9002787 19772347 PMC2761978

[58] Kazamia E, Sutak R, Paz-Yepes J, et al. Endocytosis-mediated siderophore uptake as a strategy for Fe acquisition in diatoms. *Sci Adv* 2018;4:eaar4536. 10.1126/sciadv.aar4536 29774236 PMC5955625

[59] Hoke AK, Reynoso G, Smith MR, et al. Genomic signatures of Lake Erie bacteria suggest interaction in the *Microcystis* phycosphere. *PloS One* 2021;16:e0257017. 10.1371/journal.pone.0257017 34550975 PMC8457463

[60] Clarke TA, Edwards MJ, Gates AJ, et al. Structure of a bacterial cell surface decaheme electron conduit. *Proc Natl Acad Sci U S A* 2011;108:9384–9. 10.1073/pnas.1017200108 21606337 PMC3111324

[61] Guest RL, Court EA, Waldon JL, et al. Impaired efflux of the siderophore enterobactin induces envelope stress in *Escherichia coli*. *Front Microbiol* 2019;10:2776. 10.3389/fmicb.2019.02776 31866967 PMC6908949

[62] Govindan JA, Jayamani E, Ruvkun G. ROS-based lethality of *Caenorhabditis elegans* mitochondrial electron transport mutants grown on *Escherichia coli* siderophore iron release mutants. *Proc Natl Acad Sci U S A* 2019;116:21651–8. 10.1073/pnas.1912628116 31591219 PMC6815122

[63] Fourquez M, Devez A, Schaumann A, et al. Effects of iron limitation on growth and carbon metabolism in oceanic and coastal heterotrophic bacteria. *Limnol Oceanogr* 2014;59:349–60. 10.4319/lo.2014.59.2.0349

[64] Ahn S, Jung J, Jang IA, et al. Role of glyoxylate shunt in oxidative stress response. *J Biol Chem* 2016;291:11928–38. 10.1074/jbc.M115.708149 27036942 PMC4882458

[65] Koedooder C, Guéneuguès A, Van Geersdaële R, et al. The role of the glyoxylate shunt in the acclimation to iron limitation in marine heterotrophic bacteria. *Front Mar Sci* 2018;5:435. 10.3389/fmars.2018.00435

[66] Sourice M, Askenasy I, Garcia PS, et al. A diverged transcriptional network for usage of two Fe-S cluster biogenesis machineries in the Delta-Proteobacterium *Myxococcus xanthus*. *mBio* 2023;14:e03001–22. 10.1128/mbio.03001-22 36656032 PMC9973013

[67] Chareyre S, Mandin P. Bacterial iron homeostasis regulation by sRNAs. *Microbiol Spectrum* 2018;6:RWR-0010-2017. 10.1128/microbiolspec.RWR-0010-2017 PMC11633579 29573257

[68] Touati D . Iron and oxidative stress in bacteria. *Arch Biochem Biophys* 2000;373:1–6. 10.1006/abbi.1999.1518 10620317

[69] Adler C, Corbalan NS, Peralta DR, et al. The alternative role of enterobactin as an oxidative stress protector allows *Escherichia coli* colony development. *PloS One* 2014;9:e84734. 10.1371/journal.pone.0084734 24392154 PMC3879343

[70] Qiu GW, Koedooder C, Qiu BS, et al. Iron transport in cyanobacteria – from molecules to communities. *Trends Microbiol* 2022;30:229–40. 10.1016/j.tim.2021.06.001 34175176

[71] Kranzler C, Lis H, Shaked Y, et al. The role of reduction in iron uptake processes in a unicellular, planktonic cyanobacterium. *Environ Microbiol* 2011;13:2990–9. 10.1111/j.1462-2920.2011.02572.x 21906223

[72] Conrad JC, Gibiansky ML, Jin F, et al. Flagella and pili-mediated near-surface single-cell motility mechanisms in *P. aeruginosa*. *Biophys J* 2011;100:1608–16. 10.1016/j.bpj.2011.02.020 21463573 PMC3072661

[73] Reguera G, McCarthy KD, Mehta T, et al. Extracellular electron transfer via microbial nanowires. *Nature* 2005;435:1098–101. 10.1038/nature03661 15973408

[74] Gralnick JA, Bond DR. Electron transfer beyond the outer membrane: putting electrons to rest. *Annu Rev Microbiol* 2023;77:517–39. 10.1146/annurev-micro-032221-023725 37713456

