# Supplementary material for: Photosynthesis-driven interactions in the phycosphere enhance bacterial extracellular superoxide production
Source: ISME J. 2026 Jul 5;20(1):wrag175. doi: 10.1093/ismejo/wrag175 (PMC13440305; doi:10.1093/ismejo/wrag175)
Supplement: Supplementary file 1 [file supplementary_material_wrag175.zip › Supporting_Information_wrag175.pdf]

Supporting Information for

Photosynthesis-driven interactions in the phycosphere enhance bacterial extracellular superoxide production

Xi Wang[a,b], Baiwen Ma[a,b], Hongwei Yu[a,b], Lin Su[c], Jing Qi[a,b]*, Zhugen Yang[d], Chengzhi Hu[a,b], Jiuhui Qu[a,b]*

[a] Key Laboratory of Environmental Aquatic Chemistry, State Key Laboratory of Regional Environment and Sustainability, Research Center for Eco-Environmental Sciences, Chinese Academy of Sciences, Beijing 100085, China
[b] University of Chinese Academy of Sciences, Beijing 100049, China
[c] Department of Biochemistry, School of Biological and Behavioural Sciences, Queen Mary University of London, Mile End Road, London E1 4NS, UK.
[d] Faculty of Engineering and Applied Sciences, Cranfield University, Cranfield MK43 0AL, UK

***Corresponding authors:**
Jiuhui Qu, Research Center for Eco-Environmental Sciences, Chinese Academy of Sciences, 18 Shuangqing Road, Haidian District, Beijing, BJ, 100085, China. Email: jhqu@rcees.ac.cn
Jing Qi, Research Center for Eco-Environmental Sciences, Chinese Academy of Sciences, 18 Shuangqing Road, Haidian District, Beijing, BJ, 100085, China. Email: jingqi@rcees.ac.cn

This PDF file includes: Supporting text and Figures S1 to S26

Supporting Information Text

Materials and Methods

Coculture Process Monitoring

The washed QJX-1 and *M. aeruginosa* were resuspended in sterilized BG-11 medium supplemented with 250 mg/L glucose for subsequent experiments. The final cell density of QJX-1 was adjusted to 3×10^7 cells/mL, and that of *M. aeruginosa* was adjusted to 1×10^6 cells/mL. Three treatments were established, including the *M. aeruginosa* monoculture, the QJX-1 monoculture, and the algal-bacterial coculture, with three biological replicates for each treatment. Each culture was prepared in a 500 mL Erlenmeyer flask, sealed with a gas-permeable membrane, and incubated in an artificial climate chamber. The cultivation conditions were maintained at a 12 h light / 12 h dark cycle, 29℃, 50% relative humidity, and a light intensity of 400-650 lx. These culture conditions were kept consistent throughout all experiments in this study.

At designated time points, culture supernatants were collected by centrifugation at 8000 rpm for 10 min and filtered through sterile 0.22 μm PES membranes for subsequent analyses.

Relevant Indexes of Antioxidant System. H_2O_2 content and CAT activity in the supernatant were determined using an H_2O_2 assay kit and a CAT activity assay kit (Solarbio, China), respectively. The absorbance was measured at 415 nm for H_2O_2 and 240 nm for CAT activity using a Spark 10M microplate reader (Tecan, Switzerland). GSH content in the supernatant was determined using a glutathione assay kit (BioAssay Systems, USA), with absorbance measured at 412 nm using the same microplate reader. SOD activity in the supernatant was determined using a SOD activity assay kit (LABLEAD, China), and absorbance was measured at 450 nm.

Extracellular NOX activity. Extracellular NOX activity in culture supernatants was determined using an NOX activity assay kit (Solarbio, China). In this assay, NOX catalyzes the oxidation of NADPH, which is coupled to the reduction of the artificial electron acceptor 2,6-dichlorophenolindophenol (DCPIP). Enzyme activity was quantified by continuously monitoring the decrease in absorbance at 600 nm with a Spark 10M microplate reader (Tecan, Switzerland), following the manufacturer's instructions.

Siderophore content. Siderophore content in the supernatant was determined using the universal chrome azurol S (CAS) colorimetric assay[1]. Filtered supernatants were mixed with CAS reagent at an equal volume ratio, and absorbance at 630 nm was measured after equilibrium was reached using a Spark 10M microplate reader (Tecan, Switzerland). In this assay, siderophores competitively remove Fe^{3+} from the blue CAS-Fe^{3+}-HDTMA complex, and a decrease in absorbance indicates an increase in relative siderophore content.

Glucose consumption. Glucose concentration in the supernatant was determined using a glucose quantification kit (E1010, APPLYGEN, China) based on the glucose oxidase/peroxidase method. A calibration curve relating glucose concentration to optical density at 550 nm, measured with a Spark 10M microplate reader (Tecan, Switzerland), was used to quantify glucose concentrations in the samples.

Metabolomic Analyses. Culture supernatants were collected at 48, 72, and 108 h. Samples were first centrifuged at 1000 g for 10 min at 4℃ to remove cells, and the resulting supernatants were centrifuged again at 2000 g for 10 min at 4℃ to remove residual cell fragments and precipitates. The clarified supernatants were then aliquoted, freeze-dried in a

lyophilizer, and stored at −80℃ prior to analysis. Equal aliquots of all samples were pooled to generate quality control (QC) samples, which were injected at regular intervals (every 10 samples) during the analytical run to monitor instrumental stability.

Untargeted metabolomic profiling was performed using a UHPLC-Q Exactive HF-X mass spectrometry system (Thermo Fisher Scientific, USA). Raw data were imported into Progenesis QI for peak alignment, retention time correction, and peak integration. Putative metabolite annotation was achieved by matching against public and in-house databases, including HMDB and Metlin. The resulting annotation matrix was further quality-controlled and filtered on the Majorbio Cloud Platform. The cleaned dataset was then analyzed in R using the *ropls* package to perform principal component analysis (PCA) and orthogonal partial least squares-discriminant analysis (OPLS-DA). Differential metabolites were identified using a combined threshold of Student's *t*-test ($P < 0.05$) and variable importance in projection (VIP > 1). KEGG pathway annotation and Fisher's exact test-based enrichment analysis were subsequently performed in Python using the *scipy.stats* package.

RNA Extraction, Sequencing, and Transcriptomic Analysis. Microbial samples were collected at 48 and 108 h, corresponding to the carbon-depletion and carbon-starvation stages, respectively. Samples were immediately flash-frozen in liquid nitrogen and stored at -80℃ until RNA extraction. Total RNA was extracted using TRIzol reagent (Invitrogen) and treated with DNase I (TaKaRa) to remove residual genomic DNA. RNA integrity was assessed using an Agilent 2100 Bioanalyzer, and RNA concentration and purity were measured with a NanoDrop ND-2000 spectrophotometer.

Strand-specific libraries were constructed using the TruSeq RNA Sample Preparation Kit (Illumina). Ribosomal RNA was removed with the Ribo-Zero Magnetic Kit, and the remaining RNA was fragmented into ~200 bp fragments. Double-stranded cDNA was synthesized using random primers, with dUTP incorporated during second-strand synthesis. After end repair, A-tailing, and adaptor ligation, the second strand was digested with uracil-N-glycosylase (UNG) to generate strand-specific libraries. Libraries were amplified by 15 PCR cycles, quantified using a TBS-380 fluorometer, and sequenced on a HiSeq X Ten System (Illumina) in paired-end 2×150 bp mode.

Raw sequencing data were converted to FASTQ format and quality-filtered by removing adaptor sequences, low-quality reads ($Q < 20$), reads containing >10% ambiguous bases, and reads shorter than 25 bp. Clean reads were aligned to the reference genome using Bowtie2, and residual rRNA contamination was assessed against the Rfam database. Gene expression levels were quantified with RSEM and normalized as FPKM values. Differential expression analysis was performed using edgeR, DESeq2, or DESeq. Functional enrichment of differentially expressed genes was conducted using GOATOOLS for GO analysis and KOBAS 2.0 for KEGG pathway analysis. Adjusted $P < 0.05$ was used as the threshold for statistical significance.

Analysis of extracellular redox-active components

The washed QJX-1 and *M. aeruginosa* were resuspended in sterile BG-11 medium. In the coculture, the final cell densities of QJX-1 and *M. aeruginosa* were set to 3×10^7 cells/mL and 1×10^6 cells/mL, respectively. To ensure comparability of electrochemical signals and extracellular redox-related components among treatments, algal and bacterial cell densities were determined separately by flow cytometry, and the algal monoculture, bacterial

monoculture, and algal-bacterial coculture were then adjusted to the same total cell density to generate cell-number-matched suspensions. The adjusted cultures were further incubated under the cultivation conditions described above and sampled at designated time points for photocurrent measurements and three-dimensional fluorescence analysis.

Photoelectrochemical analysis. Photocurrent measurements were performed using an electrochemical workstation coupled with a solar simulator. Current–time (*I*-*t*) curves were recorded with screen-printed electrodes (TC201, POTEN). The working electrode (WE) had an area of 0.79 mm^2, and the inter-electrode distances were 1.95 mm (WE-CE), 0.68 mm (WE-RE), and 0.25 mm (CE-RE). Illumination was provided by a solar simulator (Abet Technologies, USA) at 1000 W m^{-2}, calibrated at the height of the electrode surface in the absence of sample.

After 24 h of starvation treatment, the cultures were collected, centrifuged, washed with phosphate buffer (PB), and resuspended in PB to obtain the test suspensions. Before measurement, the suspensions were incubated in the dark for 2 h. For each test, 50 μL of sample was directly loaded onto the working electrode area of the screen-printed electrode. During measurements, the electrode was placed on a temperature-controlled platform to minimize evaporation caused by illumination. No external stirring was applied during the short-term photocurrent tests (3-10 min), and no obvious cell sedimentation was observed over the measurement period, ensuring signal stability and reproducibility.

Three-dimensional fluorescence analysis. Appropriate volumes of culture supernatants were filtered through 0.22 μm PES membranes and analyzed using a fluorescence spectrophotometer. Excitation wavelengths (Ex) were scanned from 200 to 450 nm, and emission wavelengths (Em) were recorded from 250 to 550 nm. Ultrapure water was used as a blank for background subtraction. Based on the obtained excitation-emission matrix spectra, dissolved organic matter was relatively quantified using the conventional fluorescence regional integration approach to characterize changes in extracellular organic composition during algal-bacterial interaction.

Extracellular reducing power. Extracellular NADPH and $NADP^+$ contents were determined using an NADPH/$NADP^+$ assay kit (LABLEAD, China). The assay is based on a G6PDH-mediated enzymatic cycling reaction coupled with WST-8 color development. In the reaction, oxidized $NADP^+$ is first reduced to NADPH, and the generated NADPH then reduces the water-soluble tetrazolium salt WST-8 to orange formazan in the presence of an electron-coupling reagent. Because the amount of formazan produced is linearly proportional to the NADPH concentration, NADPH/$NADP^+$ levels in the samples were quantified by measuring absorbance at 450 nm using a Spark 10M microplate reader (Tecan, Switzerland).

Enzyme-mediated $O_2^{\bullet-}$ measurement

The *M. aeruginosa* monoculture, QJX-1 monoculture, and algal-bacterial coculture were cultivated under the conditions described above and sampled at the carbon-depletion stage and the carbon-starvation stage. For each culture, samples were divided into two fractions: (1) whole-cell suspensions, in which intact cells were retained to represent the overall redox activity of the system, and (2) cell-free supernatants obtained by filtration through 0.22 μm PES membranes, which were used to assess the activity of extracellular enzymes and soluble metabolites.

Exogenous NADPH, NADH, $NADP^+$, and NAD^+ were separately added to the above fractions

at a final concentration of 200 μM to evaluate the effect of external reducing equivalents on extracellular $O_2^{\bullet-}$ production and to test the role of NAD(P)H as an electron donor for NOX-like enzymes. Diphenyleneiodonium (DPI) was added at a final concentration of 100 μM as an inhibitor of flavoprotein-dependent electron transfer. SOD was added at a final concentration of 1200 kU L^{-1} as a specific negative control for $O_2^{\bullet-}$ detection to exclude non-specific background in the chemiluminescence assay.

After addition of the corresponding reagents and thorough mixing, extracellular $O_2^{\bullet-}$ production was immediately quantified using the MCLA chemiluminescence method.

Siderophore-mediated $O_2^{\bullet-}$ measurement

QJX-1 cultured for 72 h, corresponding to the early carbon-starvation stage, were used for ligand addition experiments. Enterobactin (Solarbio, China) or EDTA stock solutions were separately added to the suspensions. For the Enterobactin treatment, the final concentration gradient was 0, 2.5, 5, 10, 20, 40, and 100 nM. For the EDTA treatment, the final concentration gradient was 0, 25, 50, 75, and 100 μM. After addition, all reaction mixtures were thoroughly mixed and incubated in the dark at 30℃ for 2 h to allow sufficient interaction between the exogenous ligands and the cells. Extracellular $O_2^{\bullet-}$ production was then immediately quantified using the MCLA chemiluminescence method described above. Each treatment and the corresponding ligand-free control were performed with three biological replicates.

In addition to QJX-1, *Pseudomonas putida* (P19) and *Bacillus subtilis* (GHSPW) were selected as test strains. Cell suspensions collected at 24, 72, and 120 h were separately amended with Enterobactin (final concentrations: 10, 20, and 40 nM) or EDTA (final concentrations: 25, 50, and 75 μM). After mixing, all samples were incubated in the dark at 30℃ for 2 h, and extracellular $O_2^{\bullet-}$ production was subsequently measured by chemiluminescence.

Effect of bacterial metabolic state. To further evaluate the role of bacterial metabolic state in siderophore-stimulated extracellular superoxide production, carbon-source replenishment experiments were conducted using bacterial suspensions collected at 120 h, corresponding to the late carbon-starvation stage. In the first set of experiments, fixed concentrations of Enterobactin or EDTA were added together with different concentrations of glucose, and the recovery of extracellular $O_2^{\bullet-}$ production was determined. In a second set of experiments, to exclude the possibility of a glucose-specific effect, Enterobactin-treated 120 h cultures were separately supplemented with 25 mg L^{-1} of conventional carbon sources (sucrose or glycerol phosphate) or key differential metabolites identified by metabolomic analysis (lysine, tyrosine, or phenylalanine). These carbon-source replenishment experiments were conducted in parallel for QJX-1, P19, and GHSPW. After incubation in the dark at 30℃ for 2 h, extracellular $O_2^{\bullet-}$ signals were measured by chemiluminescence.

Identification of the target of siderophore action. QJX-1 cultures grown to 72 h, corresponding to the mid carbon-starvation stage, were centrifuged at 8000 r min^{-1} for 10 min at 4℃ to separate cells from extracellular metabolites. The supernatant was carefully collected and passed through a sterile 0.22 μm membrane to obtain a cell-free supernatant. The cell pellet was washed three times with sterile BG-11 and then resuspended in an equal volume of carbon-free BG-11 medium to prepare whole-cell suspensions.

Whole-cell suspensions and cell-free supernatants were then separately aliquoted into sterile

tubes and subjected to the following treatments:

(1) Enterobactin treatment: Enterobactin was added to both whole-cell suspensions and cell-free supernatants to a final concentration of 20 nM;

(2) Enterobactin + carbon source treatment: in whole-cell suspensions, Enterobactin (20 nM) was added together with phenylalanine at 25 mg L^{-1};

(3) Blank control: an equal volume of sterile water was added to both whole-cell suspensions and cell-free supernatants.

All treatments were performed in triplicate. After mixing, samples were incubated in the dark at 30℃ for 2 h. Chemiluminescence signals were then measured to quantify extracellular $O_2^{\bullet-}$ production.

UV–visible spectroscopic analysis of the interaction between siderophore and cytochrome c. To assess the direct effect of siderophores on Fe-containing proteins, cytochrome *c* (Cyt *c*; Sigma) was used as a model protein. Cyt *c* was first dissolved in PBS buffer (pH 7.5) to prepare working solutions at 0, 10, 20, 50, 80, and 100 μM. Absorption spectra were recorded from 300 to 600 nm using a microplate reader, and the maximum absorbance and peak position of the Soret band were determined.

Subsequently, a 100 μM Cyt *c* working solution was mixed with Enterobactin stock solution at an equimolar ratio so that the final concentrations of both Cyt *c* and Enterobactin were 100 μM. The control group received an equal volume of PBS buffer instead of Enterobactin. All reaction mixtures were incubated in the dark at 30℃ for 2 h and then scanned over the range of 300–600 nm. Changes in Soret-band intensity and peak position were analyzed by comparing the spectra of the treatment and control groups, in order to evaluate the extent to which Enterobactin perturbed the conformation and Fe coordination environment of Cyt *c*.

Functional analysis of extracellular ROS

Degradation of sulfamethoxazole and kinetic analysis. Sulfamethoxazole (SMX) was used as a model contaminant to evaluate the environmental effect of extracellular ROS. Algal monocultures, bacterial monocultures, and algal-bacterial cocultures were established as described above, and SMX was added to a final concentration of 50 μg L^{-1}. Samples were collected at designated time points, centrifuged, and filtered through 0.22 μm membranes. SMX concentrations in the filtrates were determined by UPLC-MS/MS. Degradation kinetics were first fitted using a pseudo-first-order model. To resolve stage-dependent behavior, the incubation period was further divided into a carbon-depletion stage (0-48 h) and a carbon-starvation stage (48-120 h), and apparent degradation rate constants were fitted separately by nonlinear least squares. Interval degradation rates, pseudo-R^2, and data visualization were calculated in R (version 4.5.2).

Effect of extracellular ROS on elemental metabolic phenotypes. The effects of extracellular ROS on elemental metabolism were evaluated using BIOLOG phenotype microarray plates, including ECO (31 carbon sources), PM3 (95 nitrogen sources), and PM4 (59 phosphorus sources and 35 sulfur sources). Algal-bacterial cocultures harvested at 48 h were used for the assays. In the experimental group, superoxide dismutase (SOD) and catalase (CAT) were added at final concentrations of 200 U mL^{-1} each and incubated for at least 2 h; untreated cocultures served as controls. Samples were inoculated into the microplates and incubated in the OmniLog system at 30℃ for 96 h. Raw kinetic data were baseline-corrected, and the area under the curve (AUC) was calculated for each well.

Metabolic differences were expressed as $\log_2(AUC_{Exp} / AUC_{Ctrl})$ and visualized as heat maps. Substrate information is provided in Table S4.

Chemical sensitivity phenotype analysis. Chemical sensitivity phenotypes were analyzed using BIOLOG PM11–PM20 plates, which include antibiotics, heavy metals, chelators, and other inhibitory compounds at four concentration levels. To assess the physiological role of extracellular ROS, 48 h cocultures were treated with SOD and CAT (200 U mL^{-1} each) for at least 2 h, whereas untreated cocultures served as controls. Treated suspensions were inoculated into PM11–PM20 plates and monitored in the OmniLog system at 30℃ for 60 h. Raw data were baseline-corrected, fitted to obtain kinetic curves, and integrated as AUC values. Metabolic responses were expressed as $\log_2(AUC_{Exp} / AUC_{Ctrl})$.

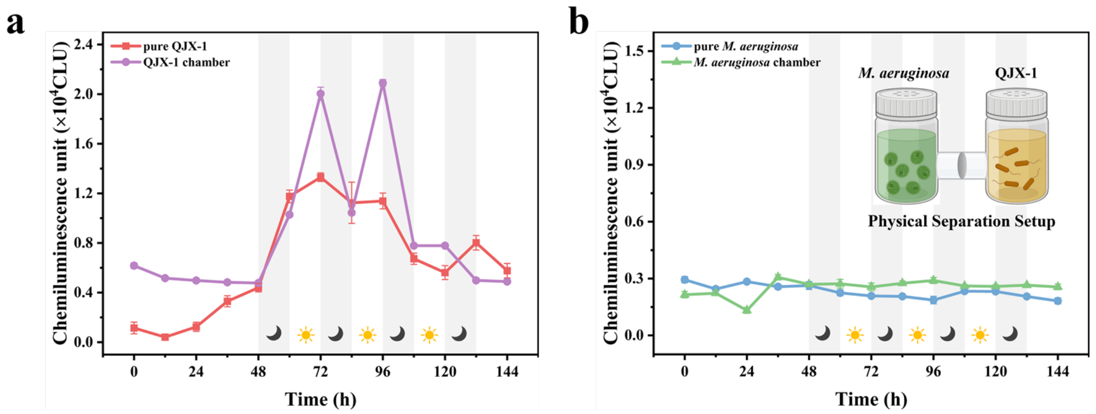

Figure S1. Extracellular $O_2^{\bullet-}$ production in monocultures and physically separated cocultures. The inset shows a schematic diagram of the physical separation setup. A 1000 kDa molecular weight cut-off membrane was used to physically separate *M. aeruginosa* and QJX-1 but allowed the exchange of diffusible substances between the two chambers. Gray and white backgrounds indicate dark and light phases, respectively.

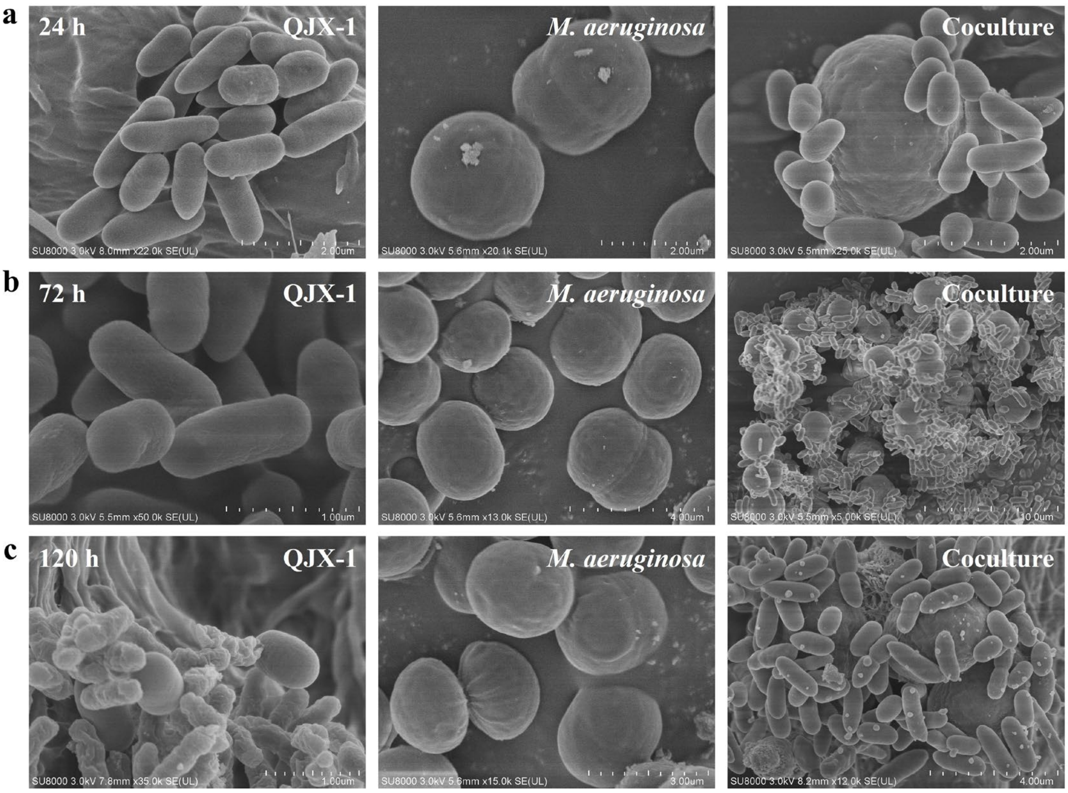

Figure S2. SEM characterization of cell morphology in monoculture and coculture at different culture stages.

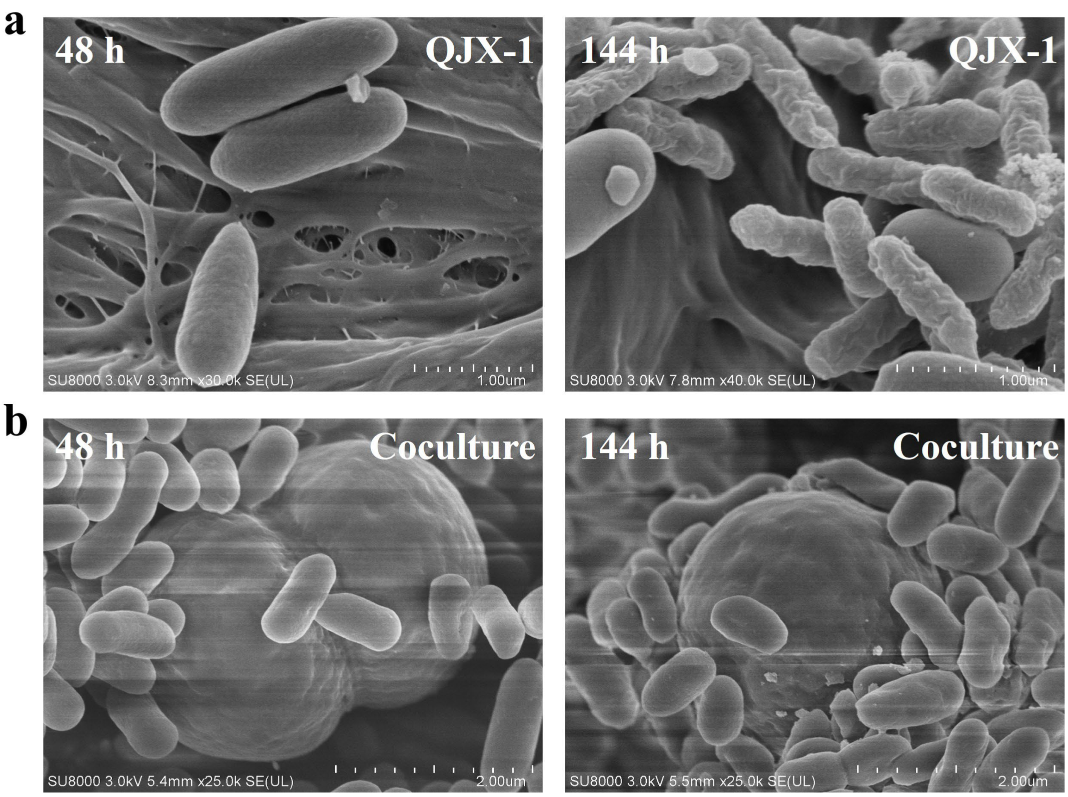

Figure S3. SEM showing morphological changes of QJX-1 in monoculture and coculture during prolonged incubation.

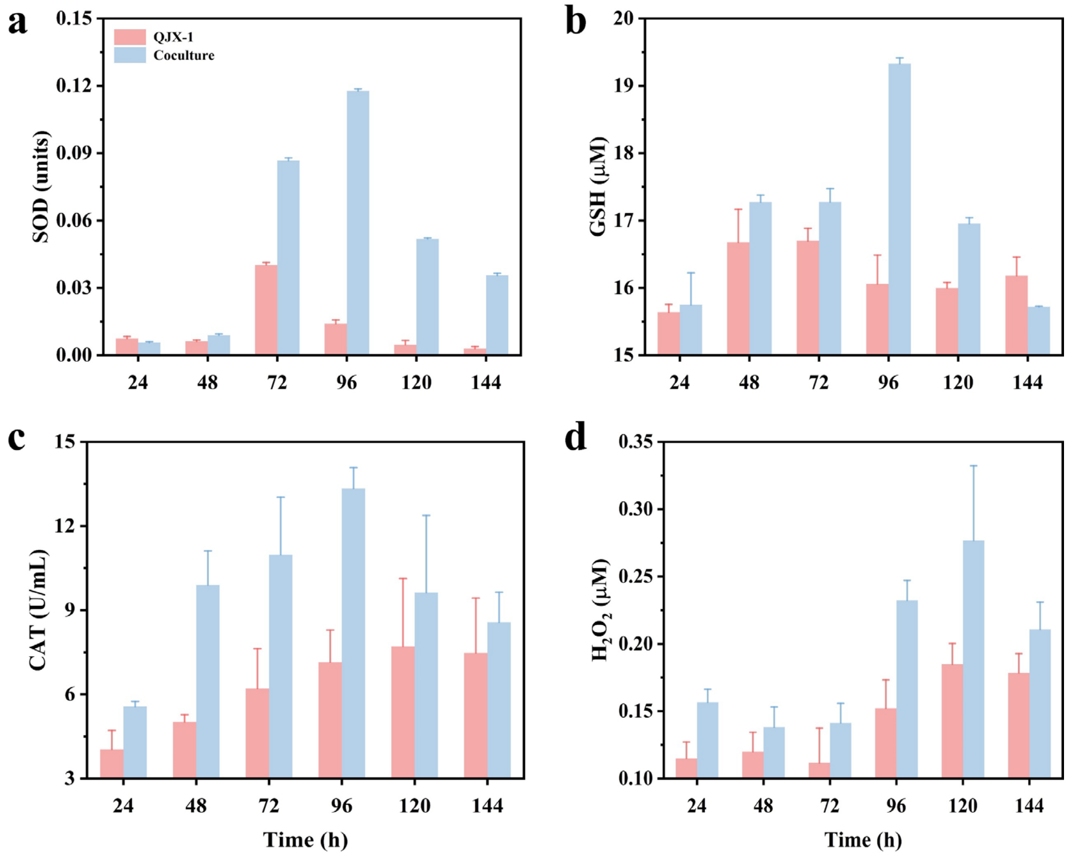

Figure S4. Temporal changes in antioxidant responses of the QJX-1 monoculture and coculture during incubation.

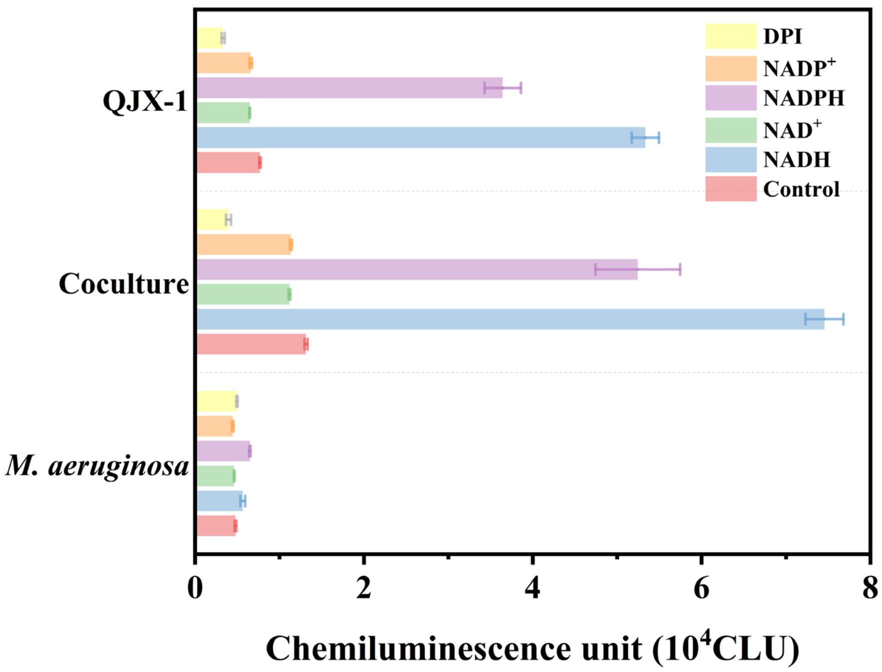

Figure S5. Effects of exogenous NADPH/NADP$^+$ and NADH/NAD$^+$ on extracellular $O_2^{\bullet -}$ production in the QJX-1 monoculture, *M. aeruginosa* monoculture, and coculture under carbon-starvation conditions. [NADPH]=200 μM, [NADP$^+$]=200 μM, [NADH]=200 μM, [NAD$^+$]=200 μM, [DPI]=100 μM.

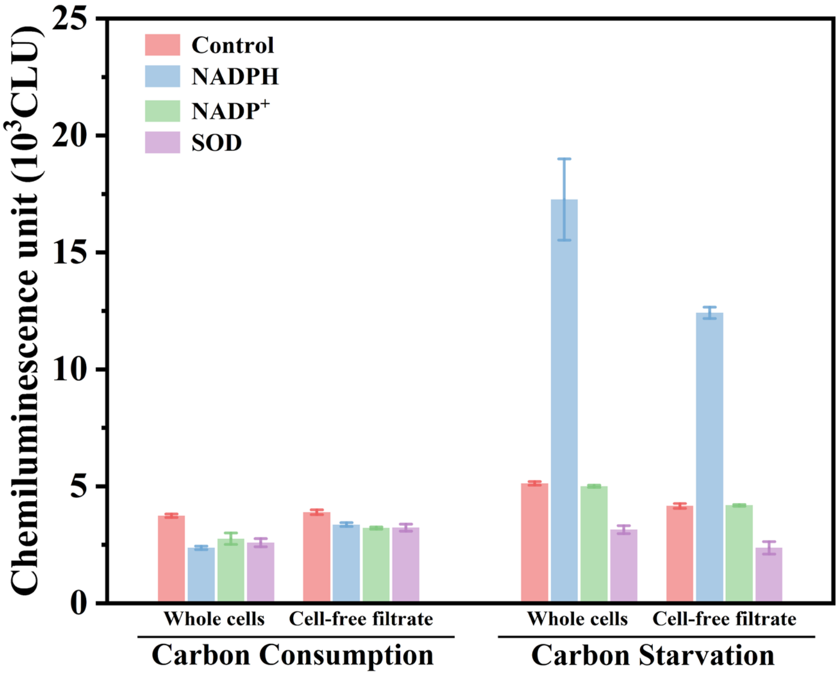

Figure S6. Effects of exogenous NADPH/NADP$^+$ on extracellular $O_2^{\bullet-}$ production in QJX-1 whole-cell suspensions and cell-free filtrates collected during the carbon-depletion and carbon-starvation phases. [NADPH]=200 μM, [NADP$^+$]=200 μM, [SOD]=1200 kU/L.

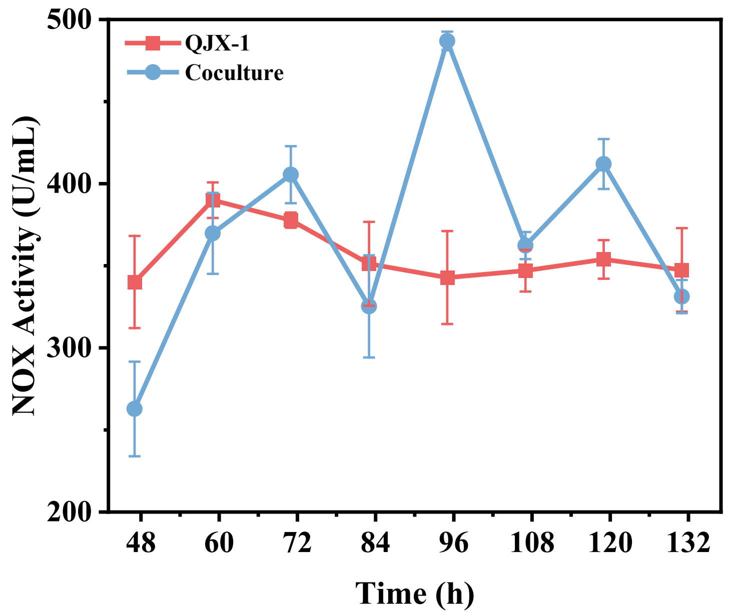

Figure S7. Temporal changes in extracellular NOX activity in the QJX-1 monoculture and coculture during incubation.

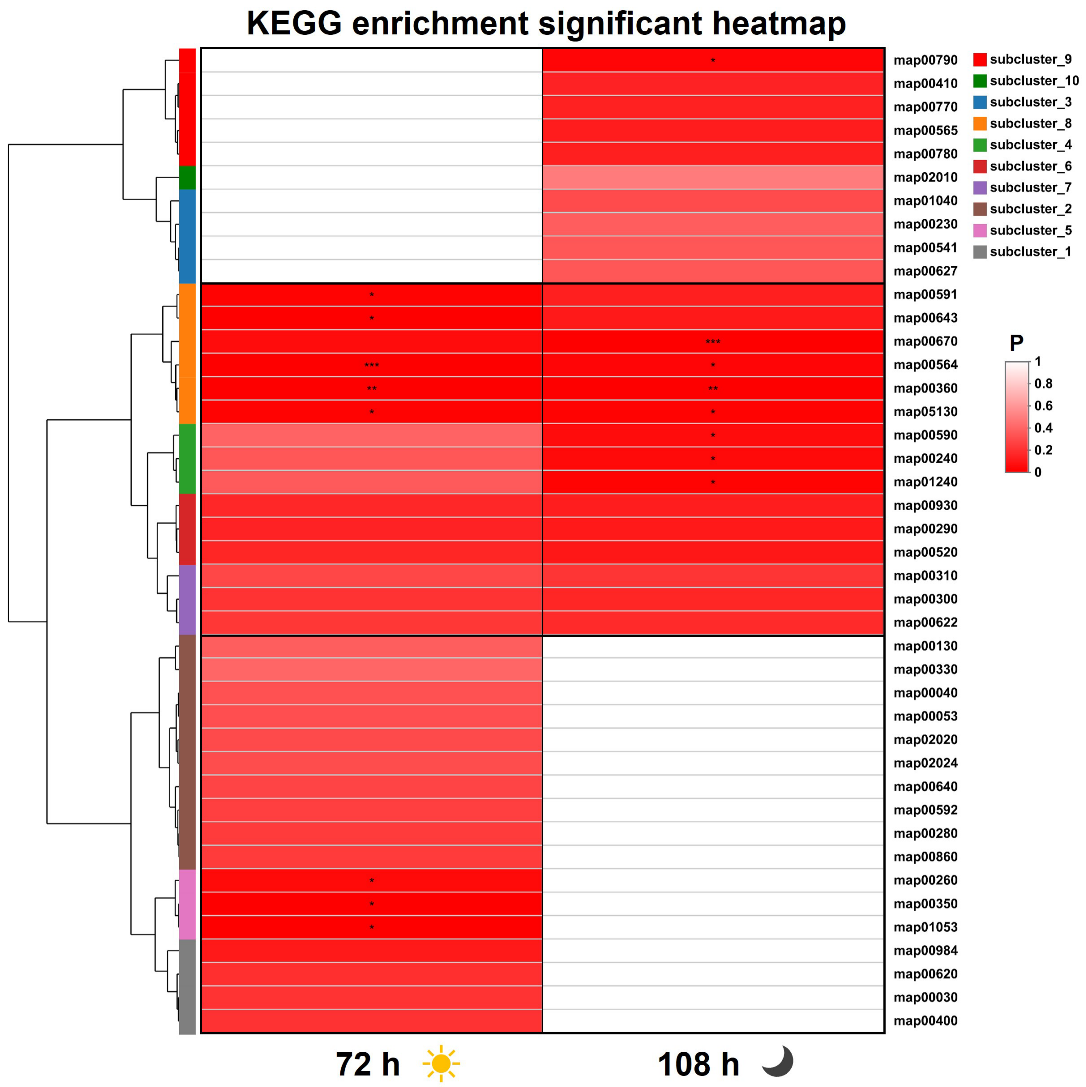

Figure S8. Hierarchical clustering of differentially enriched KEGG pathways in the coculture relative to the QJX-1 monoculture at 72 h and 108 h under light and dark conditions.

Table S1. KEGG pathway IDs and corresponding pathway descriptions for the clustered differential pathways shown in Fig. S8

Pathway ID	Pathway description	Response pattern	Significance	
			72 h	108 h
map00790	Folate biosynthesis			*
map00410	beta-Alanine metabolism			
map00770	Pantothenate and CoA biosynthesis			
map00565	Ether lipid metabolism			
map00780	Biotin metabolism	Dark-enriched		
map02010	ABC transporters			
map01040	Biosynthesis of unsaturated fatty acids			
map00230	Purine metabolism			
map00541	O-Antigen nucleotide sugar biosynthesis			
map00627	Aminobenzoate degradation			
map00591	Linoleic acid metabolism		*	
map00643	Styrene degradation		*	
map00670	One carbon pool by folate			***
map00564	Glycerophospholipid metabolism		***	*
map00360	Phenylalanine metabolism		**	**
map05130	Pathogenic *Escherichia coli* infection		*	*
map00590	Arachidonic acid metabolism	Shared background metabolism		*
map00240	Pyrimidine metabolism			*
map01240	Biosynthesis of cofactors			*
map00930	Caprolactam degradation			
map00290	Valine, leucine and isoleucine biosynthesis			
map00520	Amino sugar and nucleotide sugar metabolism			
map00310	Lysine degradation			
map00300	Lysine biosynthesis			
map00622	Xylene degradation			
map00130	Ubiquinone and other terpenoid-quinone biosynthesis			
map00330	Arginine and proline metabolism			
map00040	Pentose and glucuronate interconversions			
map00053	Ascorbate and aldarate metabolism			
map02020	Two-component system			
map02024	Quorum sensing			
map00640	Propanoate metabolism	Light-enriched		
map00592	alpha-Linolenic acid metabolism			
map00280	Valine, leucine and isoleucine degradation			
map00860	Porphyrin and chlorophyll metabolism			
map00260	Glycine, serine and threonine metabolism		*	
map00350	Tyrosine metabolism		*	
map01053	Biosynthesis of siderophore group nonribosomal peptides		*	
map00984	Steroid degradation			

Table S1 (continued)

Pathway ID	Pathway description	Response pattern	Significance	
			72 h	108 h
map00620	Pyruvate metabolism	Light-enriched		
map00030	Pentose phosphate pathway			
map00400	Phenylalanine, tyrosine and tryptophan biosynthesis			

Note: * adjusted $P < 0.05$; ** adjusted $P < 0.01$; *** adjusted $P < 0.001$

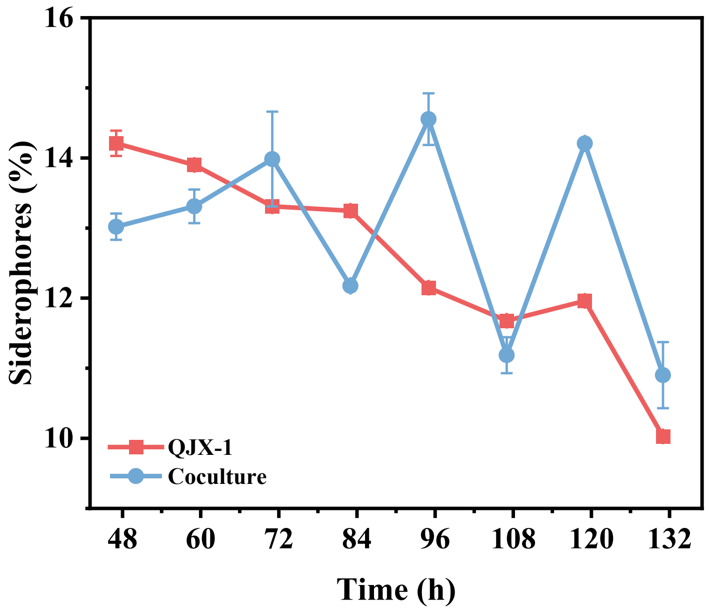

Figure S9. Temporal changes in extracellular siderophores in the QJX-1 monoculture and coculture during incubation.

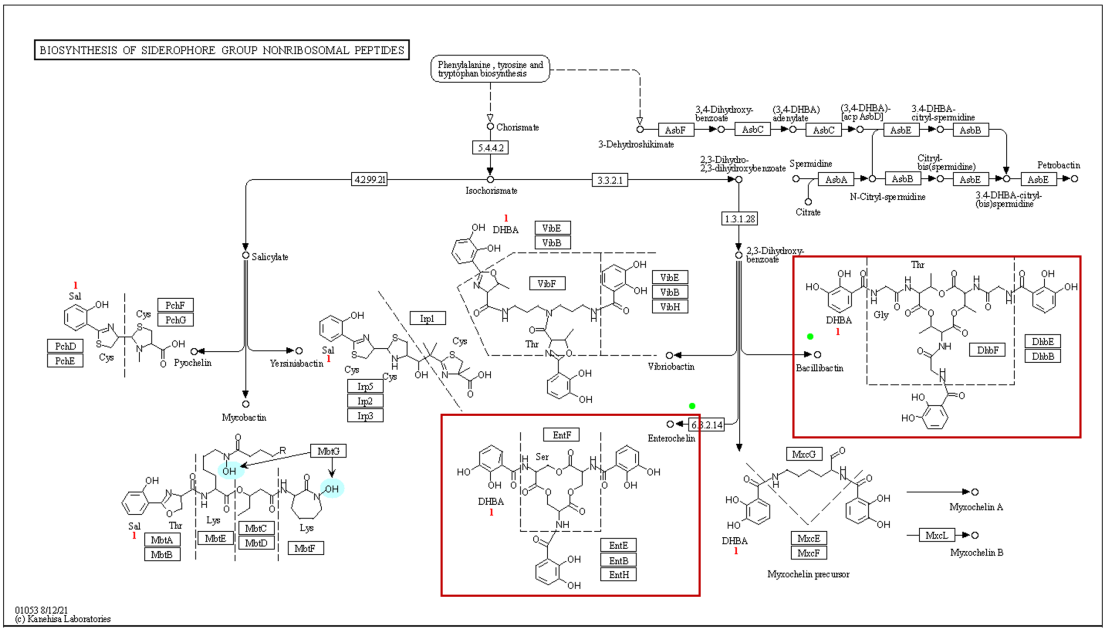

Figure S10. KEGG pathway map of chorismate-derived aromatic metabolism and catecholate siderophore biosynthesis.

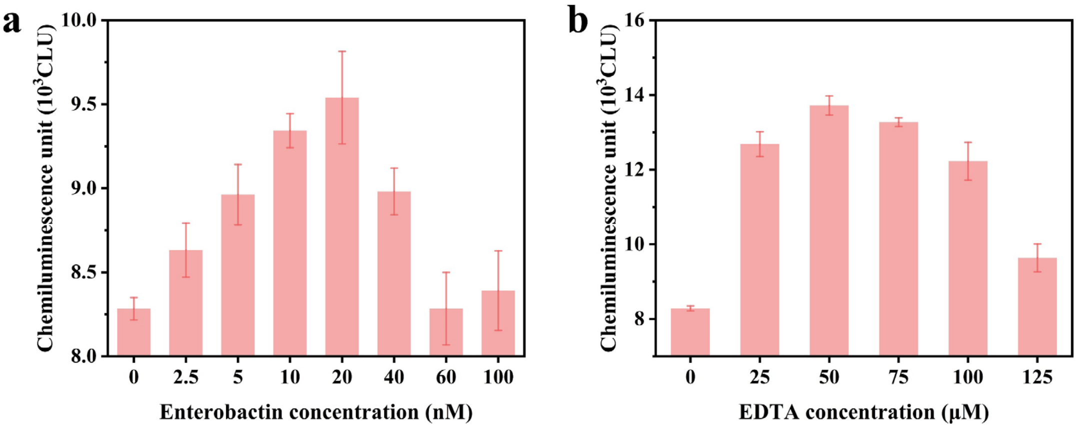

Figure S11. Effects of different Enterobactin and EDTA concentrations on extracellular $O_2^{\bullet -}$ production in QJX-1 under carbon-starvation conditions.

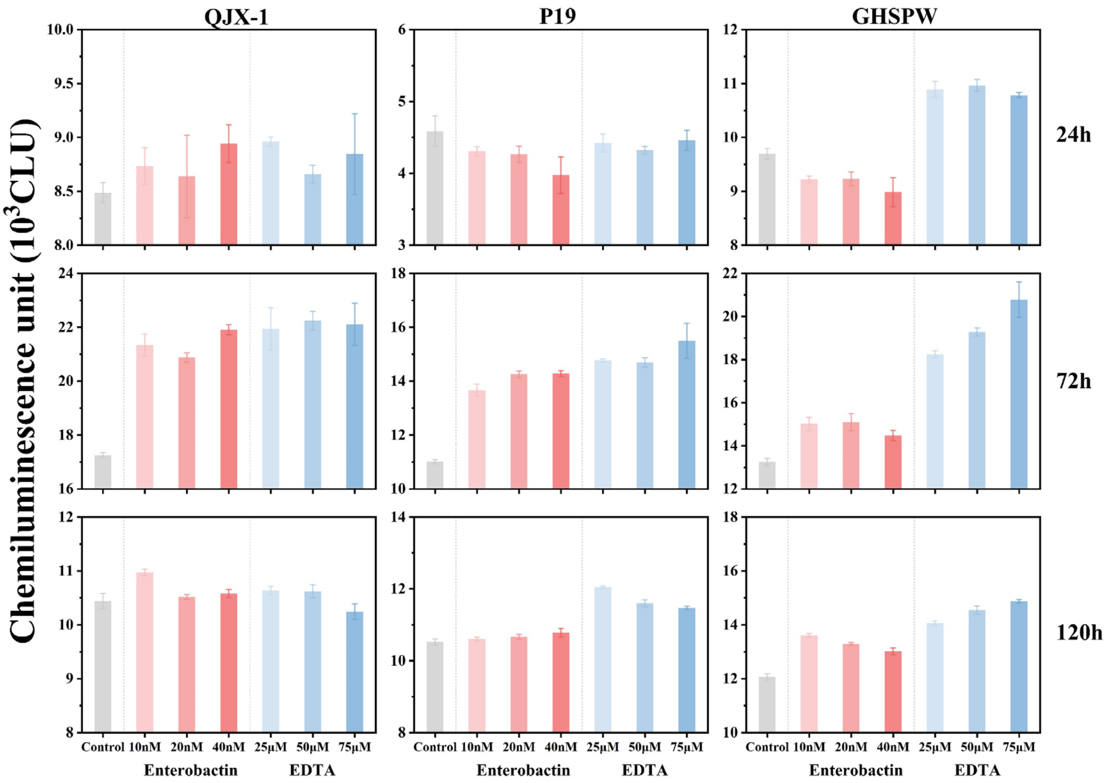

Figure S12. **Effects of Enterobactin and EDTA on extracellular $O_2^{\bullet-}$ production in heterotrophic bacteria at different incubation stages.** The heterotrophic bacteria tested were *Pseudomonas putida* strains QJX-1 and P19, and *Bacillus subtilis* strain GHSPW. Measurements were performed during the carbon-depletion (24 h), early carbon-starvation (72 h), and late carbon-starvation (120 h) phases.

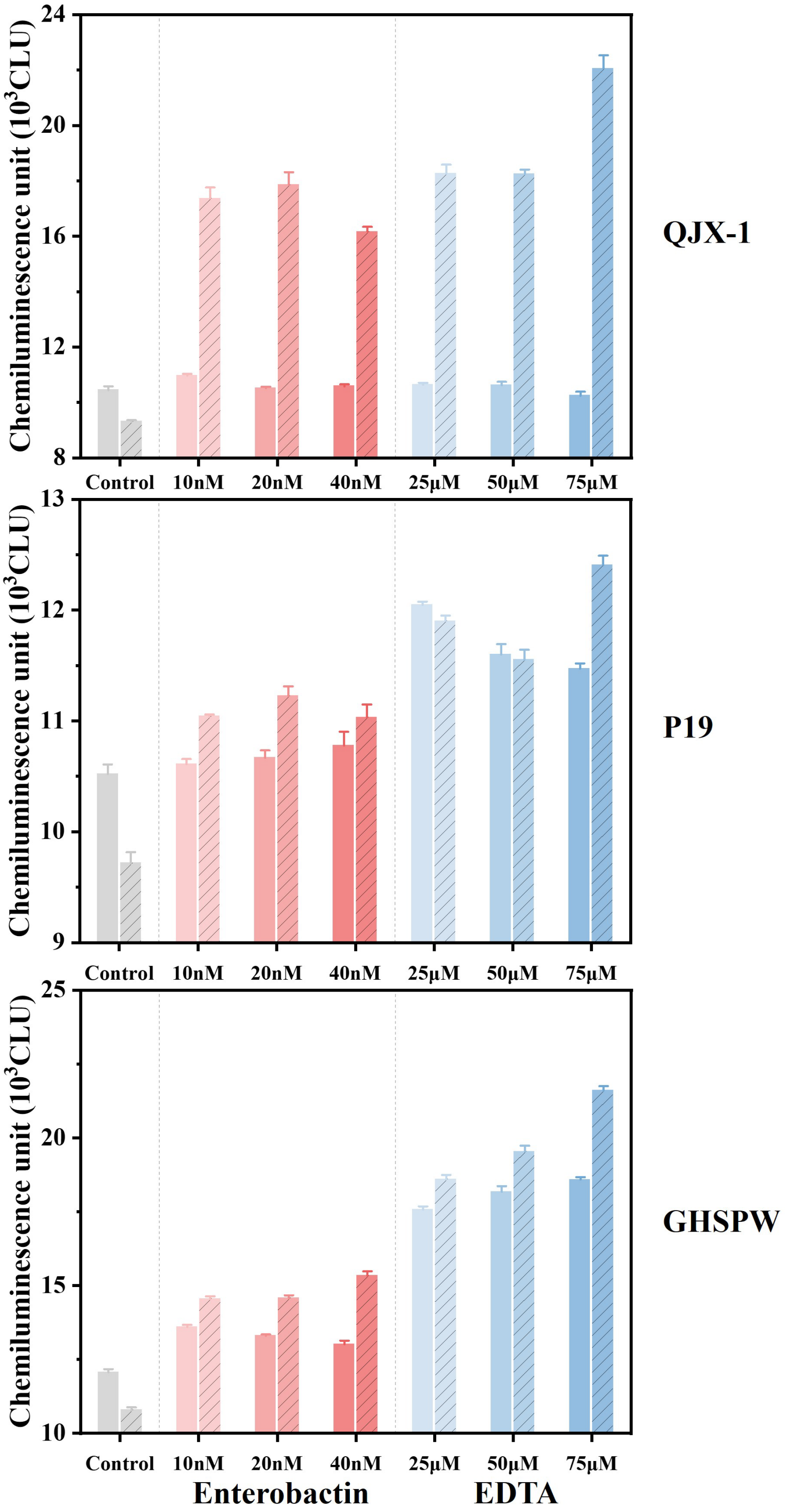

Figure S13. Effects of glucose supplementation and siderophores on extracellular $O_2^{\bullet-}$ production in heterotrophic bacteria during the late carbon-starvation phase. The heterotrophic bacteria tested were QJX-1, P19, and GHSPW. Treatments included Enterobactin and EDTA, and shaded bars indicate supplementation with 25 mg/L glucose. Measurements were performed at 120 h.

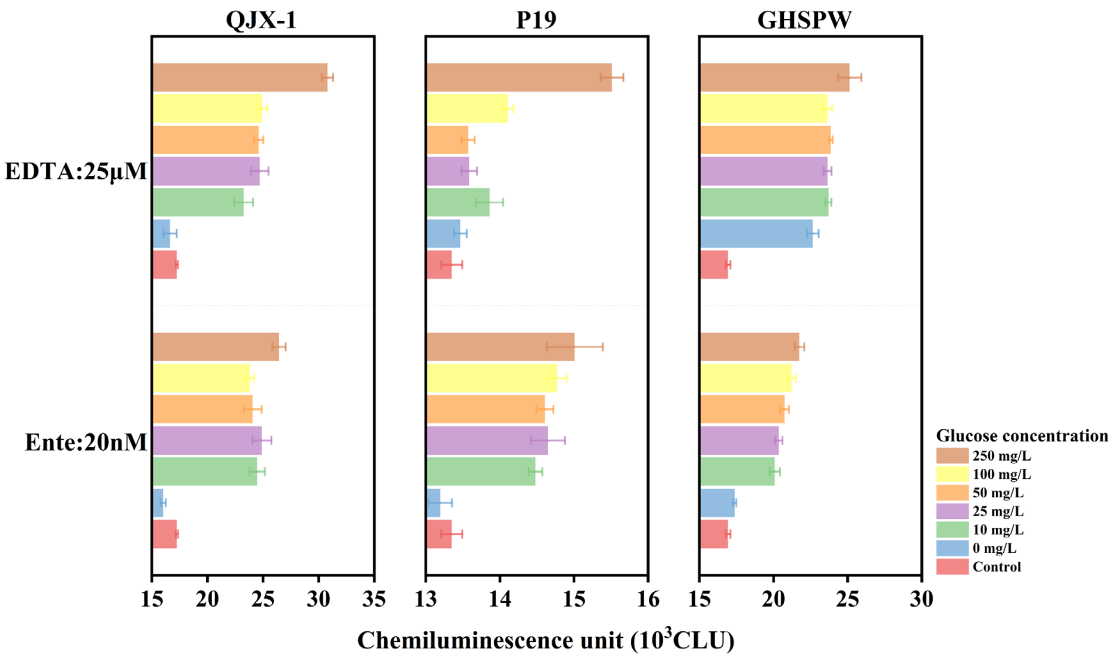

Figure S14. **Effects of different glucose concentrations on siderophore-stimulated extracellular $O_2^{\bullet-}$ production in heterotrophic bacteria during the late carbon-starvation phase.** The heterotrophic bacteria tested were QJX-1, P19, and GHSPW. Measurements were performed at 120 h. [Enterobactin]=20 nM, [EDTA]=25 μM.

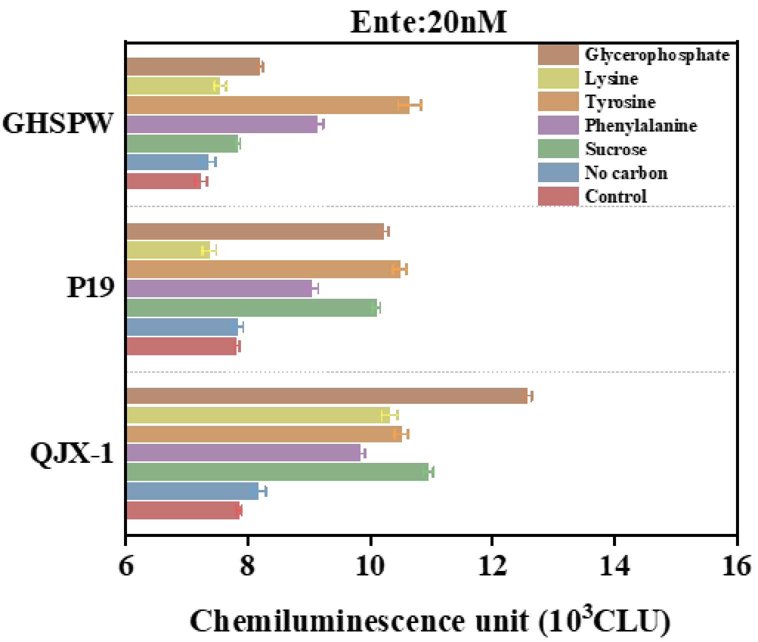

Figure S15. **Effects of different carbon sources on Enterobactin-stimulated extracellular $O_2^{\bullet-}$ production in heterotrophic bacteria during the late carbon-starvation phase.** The heterotrophic bacteria tested were QJX-1, P19, and GHSPW. Measurements were performed at 120 h. [Enterobactin]=20 nM. [Carbon sources]=25 mg/L.

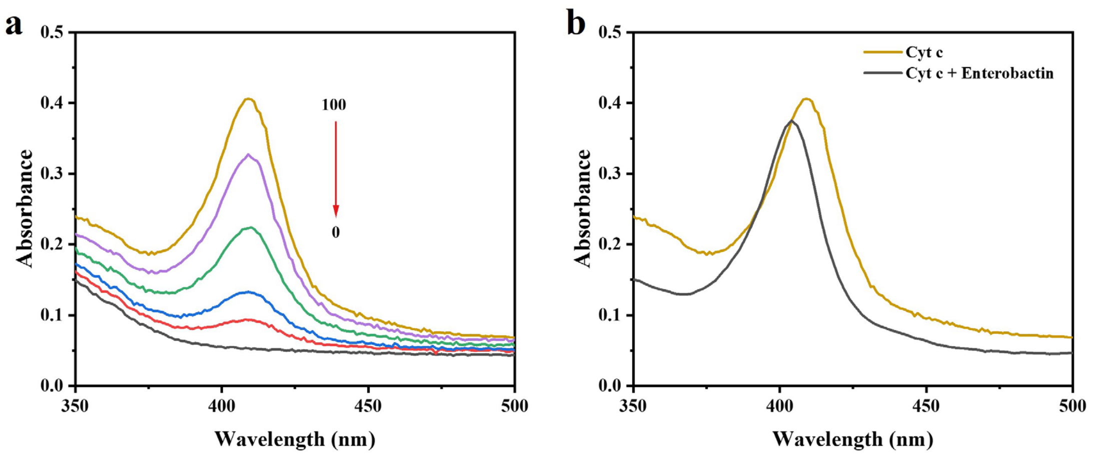

Figure S16. Soret-band spectra of cytochrome *c* and the effects of Enterobactin on its conformation. (a) Absorbance spectra of purified cytochrome *c* at concentrations ranging from 0 to 100 µM. (b) Absorbance spectra of 100 µM purified cytochrome *c* before and after incubation with 100 µM Enterobactin for 2 h.

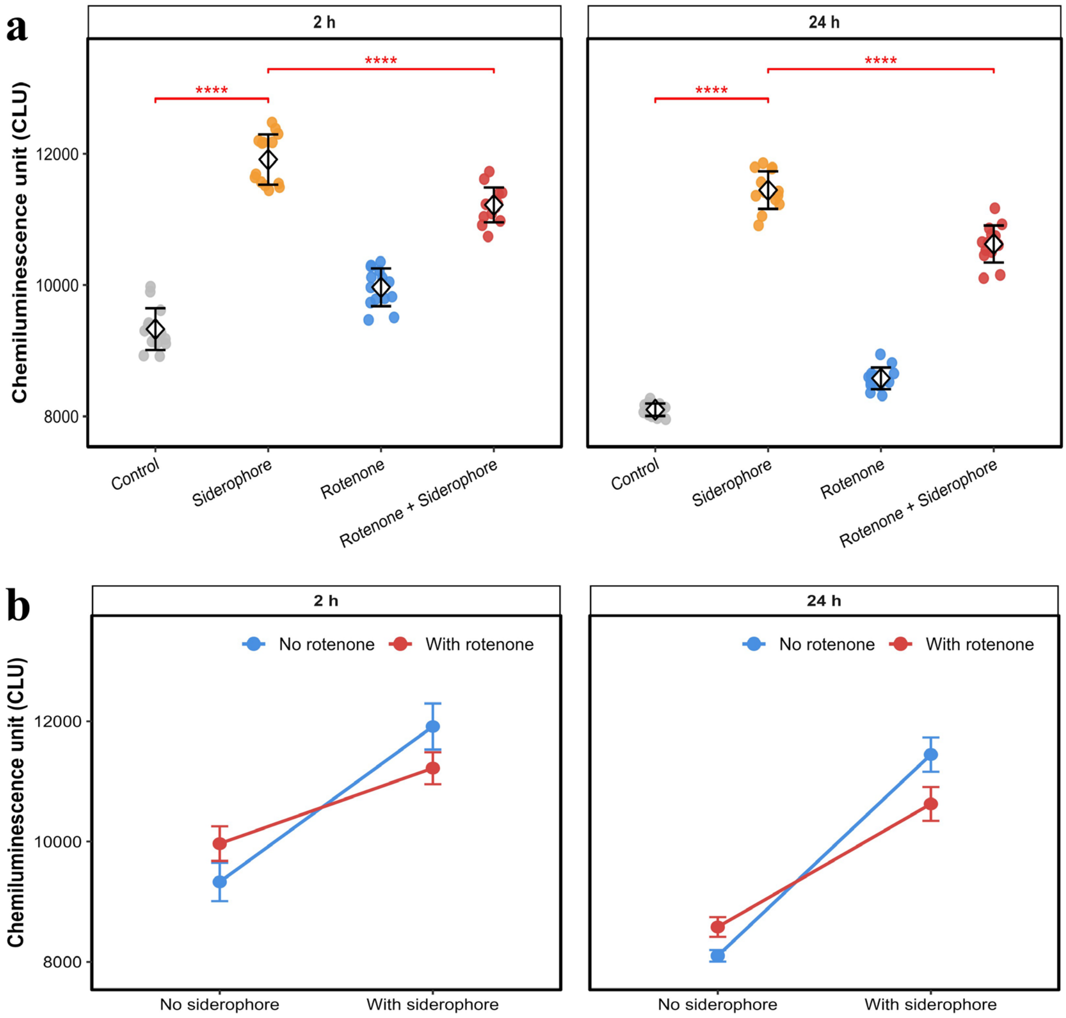

Figure S17. Rotenone attenuates siderophore-stimulated extracellular $O_2^{\bullet-}$ production in QJX-1. (a) Extracellular $O_2^{\bullet-}$ levels in QJX-1 cultures under control, siderophore (Enterobactin, 10 nM), rotenone (50 µM), and rotenone + siderophore treatments at 2 h and 24 h. Individual points represent independent replicates. Diamonds and error bars indicate mean ± SD. Siderophore significantly increased extracellular $O_2^{\bullet-}$ relative to the control, whereas rotenone significantly attenuated the siderophore-induced increase. (b) Interaction plots showing the combined effects of siderophore and rotenone on extracellular $O_2^{\bullet-}$ production at 2 h and 24 h. The non-parallel line patterns indicate that rotenone reduced the magnitude of the siderophore effect, supporting the interpretation that siderophore-enhanced extracellular $O_2^{\bullet-}$ production depends, at least in part, on electron flow through the respiratory electron transport chain.

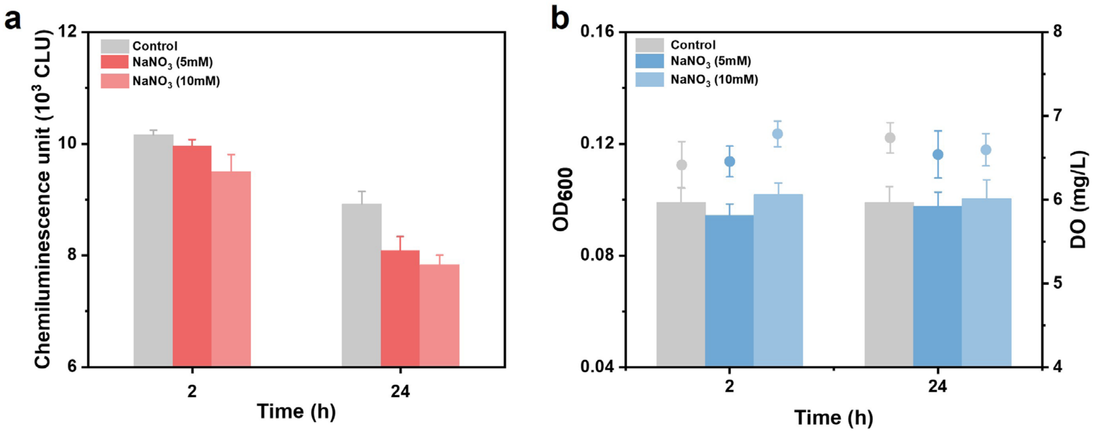

Figure S18. Effects of nitrate addition on extracellular $O_2^{\bullet-}$ production and corresponding biomass and dissolved oxygen controls. (a) Extracellular $O_2^{\bullet-}$ production in QJX-1 cultures after addition of $NaNO_3$ (0, 5, and 10 mM) in PBS at 2 h and 24 h. (b) Biomass (OD600) and dissolved oxygen (DO) measured under the same conditions. Addition of $NaNO_3$ resulted in a modest but consistent decrease in extracellular $O_2^{\bullet-}$ signal, with an overall concentration-dependent trend. No significant changes in biomass or DO were observed, indicating that the decrease in $O_2^{\bullet-}$ production was not primarily caused by toxicity or oxygen limitation.

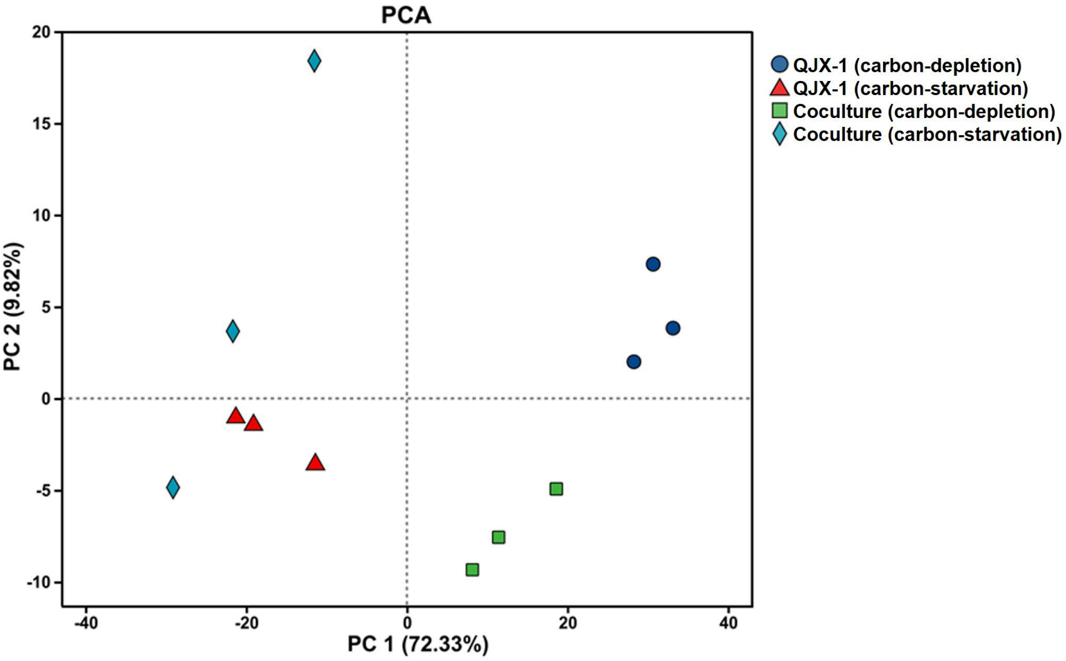

Figure S19. Principal component analysis of transcriptomic profiles in the QJX-1 monoculture and coculture during carbon depletion and carbon starvation.

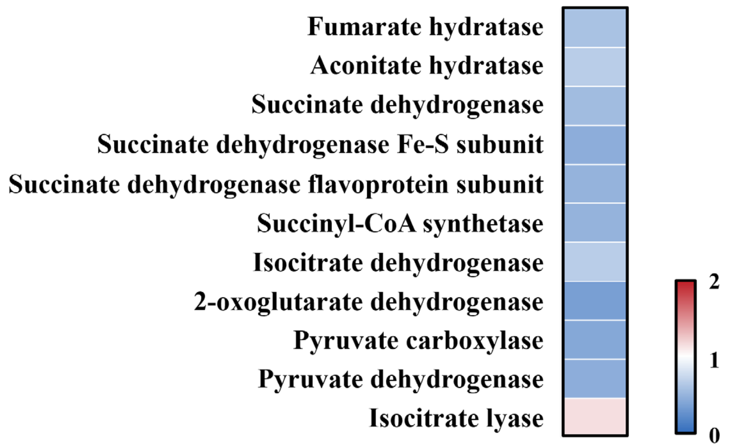

Figure S20. Relative transcript abundance of TCA cycle genes in the QJX-1 monoculture during carbon starvation relative to carbon depletion. Relative transcript abundance is expressed as FPKM (carbon-starvation) / FPKM (carbon-depletion). A value of 1 indicates no change; values > 1 indicate higher expression during carbon starvation, whereas values < 1 indicate lower expression relative to the carbon-depletion stage.

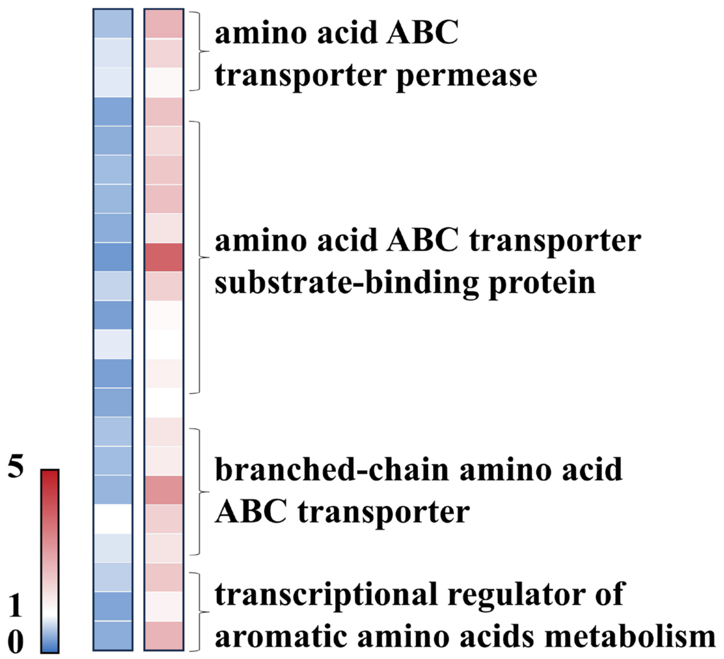

Figure S21. Relative transcript abundance of amino acid transport genes in cocultured QJX-1 relative to the QJX-1 monoculture during carbon depletion and carbon starvation. Relative transcript abundance is expressed as FPKM (Coculture) / FPKM (QJX-1 monoculture). A value of 1 indicates no change; values > 1 indicate higher expression in coculture, whereas values < 1 indicate lower expression relative to the QJX-1 monoculture. The left column represents the carbon-depletion stage and the right column represents the carbon-starvation stage.

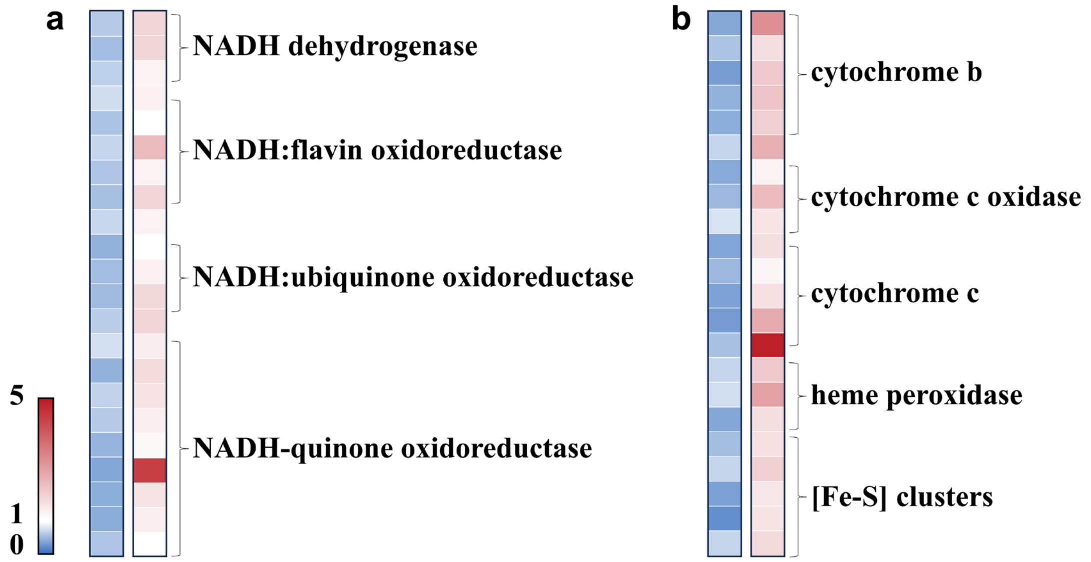

Figure S22. Relative transcript abundance of oxidative phosphorylation- and electron transport-related genes in cocultured QJX-1 relative to the QJX-1 monoculture during carbon depletion and carbon starvation. Relative transcript abundance is expressed as FPKM (Coculture) / FPKM (QJX-1 monoculture). A value of 1 indicates no change; values > 1 indicate higher expression in coculture, whereas values < 1 indicate lower expression relative to the QJX-1 monoculture. The left column represents the carbon-depletion stage and the right column represents the carbon-starvation stage.

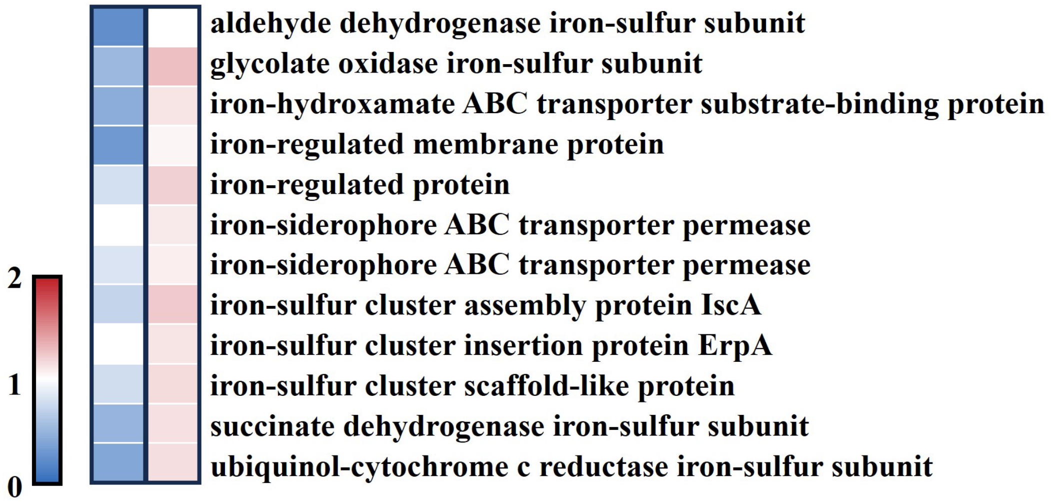

Figure S23. Relative transcript abundance of siderophore transport- and Fe-containing protein-related genes in cocultured *M. aeruginosa* relative to the *M. aeruginosa* monoculture during carbon depletion and carbon starvation. Relative transcript abundance is expressed as FPKM (Coculture) / FPKM (*M. aeruginosa* monoculture). A value of 1 indicates no change; values > 1 indicate higher expression in coculture, whereas values < 1 indicate lower expression relative to the *M. aeruginosa* monoculture. The left column represents the carbon-depletion stage and the right column represents the carbon-starvation stage.

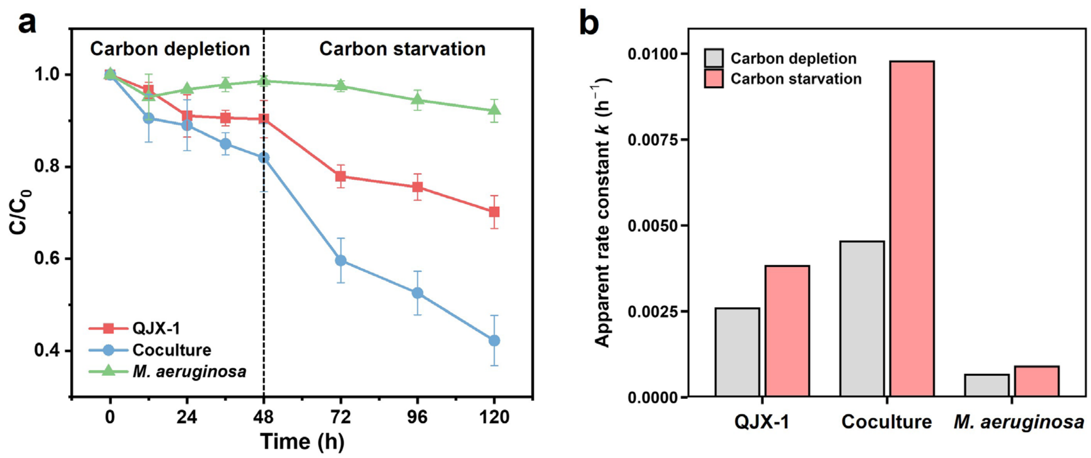

Figure S24. Degradation profiles of sulfamethoxazole (SMX) in monoculture and coculture systems and the corresponding stage-specific kinetic characteristics. (a) Time-course removal of SMX in the *M. aeruginosa* monoculture, QJX-1 monoculture, and *M. aeruginosa*–QJX-1 coculture. The dashed line indicates the transition point used to divide the degradation process into the carbon-depletion and carbon-starvation stages. (b) Apparent first-order rate constants (k) for SMX degradation during the carbon-depletion and carbon-starvation stages in the different culture systems.

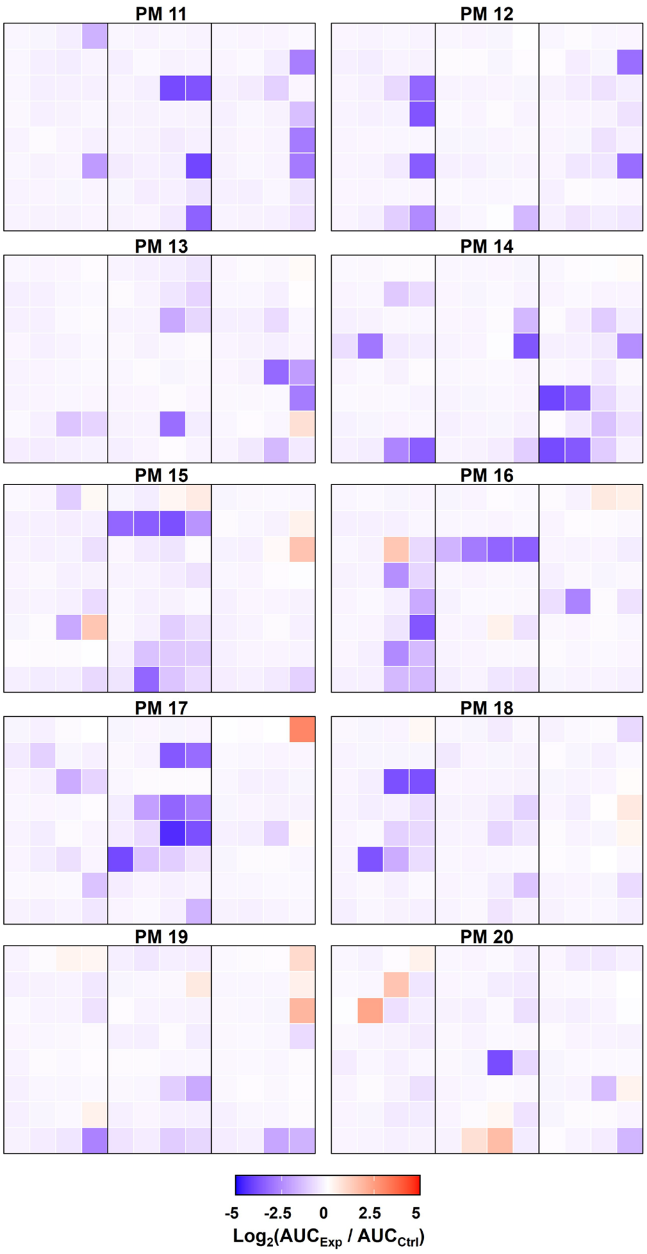

Figure S25. Heat map of AUC ratios for the algal–bacterial coculture on BIOLOG PM11–PM20 chemical sensitivity phenotype microarrays after ROS quenching. PM11–PM20 plates contain diverse inhibitory compounds, including antibiotics, heavy metals, chelators, and other metabolic inhibitors, each tested at four increasing concentrations. The natural coculture retaining extracellular ROS served as the control group (Ctrl), and the ROS-quenched coculture served as the experimental group (Exp). For each well, metabolic activity was quantified as the area under the curve (AUC), and the heat map was generated using $\log_2(AUC_{Exp} / AUC_{Ctrl})$ Red indicates enhanced metabolic activity, blue indicates reduced metabolic activity, and white indicates little or no change after ROS quenching. The predominance of blue indicates that ROS quenching generally suppressed metabolic activity across most chemical stress conditions, highlighting the importance of extracellular ROS in maintaining chemical stress tolerance in the algal–bacterial coculture.

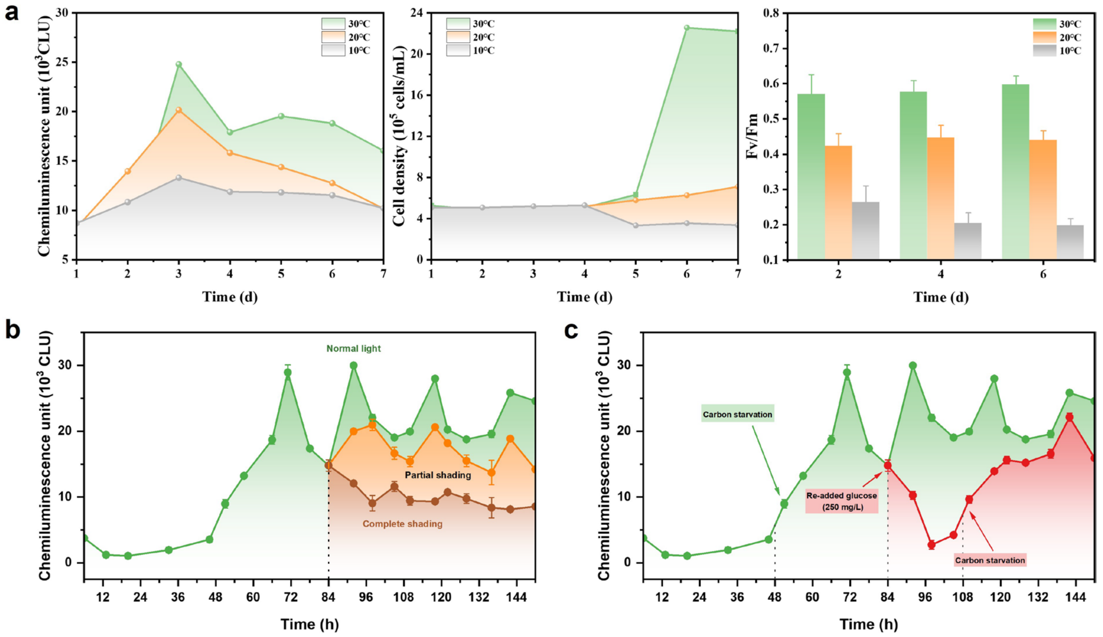

Figure S26. Temperature, light availability, and carbon status jointly regulate extracellular $O_2^{•-}$ production in the algal-bacterial coculture. (a) Effects of temperature (30, 20, and 10℃) on extracellular $O_2^{•-}$ production, algal growth, and photosynthetic performance in the coculture. (b) Effect of shading on extracellular $O_2^{•-}$ production after the coculture entered the high-ROS phase under carbon starvation. (c) Effect of glucose re-addition on extracellular $O_2^{•-}$ production during the starvation-associated high-ROS phase. Data are shown as mean ± s.d. from 3 independent biological replicates.
